# Supplementary material for: Effectiveness of mRNA boosters after homologous primary series with BNT162b2 or ChAdOx1 against symptomatic infection and severe COVID-19 in Brazil and Scotland: A test-negative design case–control study
Source: PLoS Med. 2023 Jan 11;20(1):e1004156. doi: 10.1371/journal.pmed.1004156 (PMC9879484; doi:10.1371/journal.pmed.1004156)
Supplement: S1 Appendix — Table A. Additional population characteristics. Table B. Conditions of QCovid risk algorithm. Table C. Vaccination status of individuals tested for SARS-CoV-2 in Brazil (A) and Scotland (B), according to the test result and severity of disease. Vaccinees data were detailed according to the time after each dose. Table D. Time interval in days—median (interquartile interval)—between vaccination and test in Brazil and Scotland, according to the type of vaccine used in the primary series. Table E. Vaccine effectiveness against symptomatic infection in Brazil and Scotland, expressed in percentages (95% CI), according to the type of vaccine used at the primary series and by age group. Reference group: individuals unvaccinated. Table F. Vaccine effectiveness against severe outcomes in Brazil and Scotland, expressed in percentages (95% CI), according to the type of vaccine used at the primary series and by age group. Reference group: individuals unvaccinated. Table G. Relative vaccine effectiveness against symptomatic infection in Brazil and Scotland expressed in percentages (95% CI) according to the type of vaccine used at the primary series and by age group. Reference group: individuals that received only a primary series. Table H. Relative vaccine effectiveness against severe disease in Brazil and Scotland expressed in percentages (95% CI) according to the type of vaccine used at the primary series and by age group. Reference group: individuals that received only a primary series. Table I. Vaccine effectiveness against symptomatic infection among individuals with a previously confirmed infection in Brazil compared to unvaccinated. Results were reported as percentages (95% CI), according to the type of vaccine used in the primary series and by age group. Fig A. Distribution of variants of concern in Brazil and Scotland over time. Brazil (A) and Scotland (B). Fig B. STROBE flowchart of the study population in Brazil (A) and Scotland (B). Fig C. Distribution case and contro [file pmed.1004156.s001.docx]

Contents

[Table A Additional population characteristics 3](#_Toc122295166)

[Table B: Conditions of QCovid risk algorithm 7](#_Toc122295167)

[Table C. Vaccination Status of individuals tested for SARS-CoV-2 in Brazil (A) and Scotland (B), according to the test result and severity of disease. Vaccinees data was detailed according to the time after each dose. 8](#_Toc122295168)

[Table D. Time interval in days - median (Interquartile Interval) - between vaccination and test in Brazil and Scotland, according to the type of vaccine used in the primary series. 10](#_Toc122295169)

[Table E. Vaccine Effectiveness against symptomatic infection in Brazil and Scotland, expressed in percentages (95% CI), according to the type of vaccine used at the primary series and by age group. Reference group: individuals unvaccinated. 12](#_Toc122295170)

[Table F. Vaccine Effectiveness against severe outcomes in Brazil and Scotland, expressed in percentages (95% CI), according to the type of vaccine used at the primary series and by age group. Reference group: individuals unvaccinated 15](#_Toc122295171)

[Table G. Relative Vaccine Effectiveness against symptomatic infection in Brazil and Scotland expressed in percentages (95% CI) according to the type of vaccine used at the primary series and by age group. Reference group: individuals that received only a primary series. 17](#_Toc122295172)

[Table H. Relative Vaccine Effectiveness against severe disease in Brazil and Scotland expressed in percentages (95% CI) according to the type of vaccine used at the primary series and by age group. Reference group: individuals that received only a primary series. 20](#_Toc122295173)

[Table I. Vaccine Effectiveness against symptomatic infection among individuals with a previously confirmed infection in Brazil compared to unvaccinated. Results were reported as percentages (95% CI), according to the type of vaccine used in the primary series and by age group. 21](#_Toc122295174)

[Fig A. Distribution of variants of concern in Brazil and Scotland over time 22](#_Toc122295175)

[A) Brazil 22](#_Toc122295176)

[B) Scotland 22](#_Toc122295177)

[Fig B. STROBE Flowchart of the study population in Brazil (A) and Scotland (B) 23](#_Toc122295178)

[A) Brazil 23](#_Toc122295179)

[B) Scotland 24](#_Toc122295180)

[Fig C. Distribution case and control over time in each country for individuals unvaccinated or vaccinated with ChAdOx1 or BNT162b2 as primary series. 25](#_Toc122295181)

[A) Brazil 25](#_Toc122295182)

[B) Scotland 26](#_Toc122295183)

[Fig D. Uptake of each dose, including booster dose, in individuals vaccinated with ChAdOx1 or BNT162b2 as primary series. Vaccination numbers in Brazil (A) and Scotland (B), stratified by age group and primary series. Different Y-axis scales in each age group. 27](#_Toc122295184)

[A) Brazil 27](#_Toc122295185)

[B) Scotland 29](#_Toc122295186)

[References 30](#_Toc122295187)

# Table A Additional population characteristics

1. Brazil

| **Characteristic – n(%)** | **Cases, N = 3,011,812** | **Controls, N = 2,269,774** |
| --- | --- | --- |
| **Race** |  |  |
| White | 1,311,165 (43.5) | 949,915 (41.9) |
| Black | 105,123 (3.5) | 96,322 (4.2) |
| Asian | 57,979 (1.9) | 47,649 (2.1) |
| Mixed | 924,102 (30.7) | 757,016 (33.4) |
| Indigenous | 1,504 (0.0) | 1,219 (0.1) |
| (Missing) | 611,939 (20.3) | 417,653 (18.4) |
| **Residence in capital** | 669,125 (22.2) | 610,749 (26.9) |
| **State** |  |  |
| Rondônia | 52,035 (1.7) | 35,063 (1.5) |
| Acre | 17,382 (0.6) | 12,149 (0.5) |
| Amazonas | 43,099 (1.4) | 30,778 (1.4) |
| Roraima | 8,412 (0.3) | 8,462 (0.4) |
| Pará | 41,730 (1.4) | 29,349 (1.3) |
| Amapá | 6,551 (0.2) | 2,606 (0.1) |
| Tocantins | 19,180 (0.6) | 13,916 (0.6) |
| Maranhão | 28,996 (1.0) | 18,511 (0.8) |
| Piauí | 27,727 (0.9) | 14,274 (0.6) |
| Ceará | 73,086 (2.4) | 74,423 (3.3) |
| Rio Grande do Norte | 48,707 (1.6) | 35,347 (1.6) |
| Paraíba | 49,509 (1.6) | 38,489 (1.7) |
| Pernambuco | 89,903 (3.0) | 89,608 (3.9) |
| Alagoas | 19,014 (0.6) | 16,098 (0.7) |
| Sergipe | 20,966 (0.7) | 17,190 (0.8) |
| Bahia | 110,186 (3.7) | 65,130 (2.9) |
| Minas Gerais | 395,013 (13.1) | 244,334 (10.8) |
| Espírito Santo | 1,419 (0.0) | 1,098 (0.0) |
| Rio de Janeiro | 304,019 (10.1) | 303,169 (13.4) |
| São Paulo | 671,732 (22.3) | 493,808 (21.8) |
| Paraná | 10,036 (0.3) | 6,567 (0.3) |
| Santa Catarina | 243,703 (8.1) | 170,327 (7.5) |
| Rio Grande do Sul | 440,538 (14.6) | 338,634 (14.9) |
| Mato Grosso do Sul | 69,106 (2.3) | 59,321 (2.6) |
| Mato Grosso | 49,535 (1.6) | 29,050 (1.3) |
| Goiás | 135,544 (4.5) | 94,414 (4.2) |
| Distrito Federal | 34,684 (1.2) | 27,659 (1.2) |
| **Diabetes** | 75,849 (2.5) | 63,285 (2.8) |
| **Obesity** | 27,087 (0.9) | 22,143 (1.0) |
| **Immunosuppression** | 14,765 (0.5) | 12,745 (0.6) |
| **Chronic respiratory disease** | 64,934 (2.2) | 66,748 (2.9) |
| **Cardiac disease** | 126,302 (4.2) | 106,595 (4.7) |
| **Chronic Kidney Disease** | 7,387 (0.2) | 5,154 (0.2) |
| **Healthcare Worker** | 338,048 (11.2) | 247,279 (10.9) |

1. Scotland

| **Characteristic – n(%)** |  | **Cases, N = 352,015** | **Controls, N = 203,810** |
| --- | --- | --- | --- |
| **Number of tests** | 0 | 79,964 (22.7) | 36,876 (18.1) |
|  | 1 | 84,535 (24.0) | 44,890 (22.0) |
|  | 2 | 64,260 (18.3) | 38,442 (18.9) |
|  | 3 | 42,466 (12.1) | 27,403 (13.4) |
|  | 4 | 26,039 (7.4) | 18,122 (8.9) |
|  | 5-9 | 35,443 (10.1) | 26,980 (13.2) |
|  | 10-19 | 7,200 (2.0) | 5,099 (2.5) |
|  | 20+ | 12,108 (3.4) | 5,998 (2.9) |
| **Number of risk groups** | 0 | 226,394 (64.3) | 122,253 (60.0) |
|  | 1 | 94,752 (26.9) | 58,481 (28.7) |
|  | 2 | 23,396 (6.6) | 16,809 (8.2) |
|  | 3 | 5,460 (1.6) | 4,293 (2.1) |
|  | 4 | 1,372 (0.4) | 1,305 (0.6) |
|  | 5+ | 641 (0.2) | 669 (0.3) |
| **Atrial fibrillation** | No | 349,251 (99.2) | 201,907 (99.1) |
|  | Yes | 2,764 (0.8) | 1,903 (0.9) |
| **Asthma** | No | 296,881 (84.3) | 167,891 (82.4) |
|  | Yes | 55,134 (15.7) | 35,919 (17.6) |
| **Cancer of the blood or bone marrow at any stage of treatment** | No | 350,955 (99.7) | 203,037 (99.6) |
|  | Yes | 1,060 (0.3) | 773 (0.4) |
| **Heart failure** | No | 350,888 (99.7) | 203,024 (99.6) |
|  | Yes | 1,127 (0.3) | 786 (0.4) |
| **Cerebral palsy** | No | 351,716 (99.9) | 203,580 (99.9) |
|  | Yes | 299 (0.1) | 230 (0.1) |
| **Coronary heart disease** | No | 345,666 (98.2) | 199,387 (97.8) |
|  | Yes | 6,349 (1.8) | 4,423 (2.2) |
| **Cirrhosis of the liver** | No | 350,817 (99.7) | 202,996 (99.6) |
|  | Yes | 1,198 (0.3) | 814 (0.4) |
| **Congenital heart disease or had surgery for it in the past** | No | 349,382 (99.3) | 202,147 (99.2) |
|  | Yes | 2,633 (0.7) | 1,663 (0.8) |
| **Chronic obstructive pulmonary disease (COPD)** | No | 348,070 (98.9) | 200,288 (98.3) |
|  | Yes | 3,945 (1.1) | 3,522 (1.7) |
| **Dementia** | No | 351,812 (99.9) | 203,637 (99.9) |
|  | Yes | 203 (0.1) | 173 (0.1) |
| **Diabetes Mellitus** | No | 350,117 (99.5) | 202,545 (99.4) |
|  | Yes | 1,898 (0.5) | 1,265 (0.6) |
| **Diabetes Mellitus 2** | No | 341,082 (96.9) | 196,916 (96.6) |
|  | Yes | 10,933 (3.1) | 6,894 (3.4) |
| **Epilepsy** | No | 347,862 (98.8) | 201,146 (98.7) |
|  | Yes | 4,153 (1.2) | 2,664 (1.3) |
| **A prior fracture of hip, wrist, spine or humerus** | No | 339,351 (96.4) | 195,953 (96.1) |
|  | Yes | 12,664 (3.6) | 7,857 (3.9) |
| **Motor neuron disease, multiple sclerosis, myasthenia, or Huntingtons’s Chorea** | No | 350,735 (99.6) | 203,086 (99.6) |
|  | Yes | 1,280 (0.4) | 724 (0.4) |
| **Parkinson’s disease** | No | 351,851 (100.0) | 203,692 (99.9) |
|  | Yes | 164 (0.0) | 118 (0.1) |
| **Pulmonary hypertension or pulmonary fibrosis** | No | 351,804 (99.9) | 203,648 (99.9) |
|  | Yes | 211 (0.1) | 162 (0.1) |
| **Cystic fibrosis or bronchiectasis or alveolitis** | No | 351,007 (99.7) | 202,947 (99.6) |
|  | Yes | 1,008 (0.3) | 863 (0.4) |
| **Peripheral vascular disease** | No | 351,045 (99.7) | 202,998 (99.6) |
|  | Yes | 970 (0.3) | 812 (0.4) |
| **Rheumatoid arthritis or SLE** | No | 349,214 (99.2) | 201,841 (99.0) |
|  | Yes | 2,801 (0.8) | 1,969 (1.0) |
| **Lung or oral cancer** | No | 351,735 (99.9) | 203,559 (99.9) |
|  | Yes | 280 (0.1) | 251 (0.1) |
| **Severe mental illness** | No | 310,578 (88.2) | 174,027 (85.4) |
|  | Yes | 41,437 (11.8) | 29,783 (14.6) |
| **Sickle cell disease or severe combined immune deficiency syndromes** | No | 351,763 (99.9) | 203,642 (99.9) |
|  | Yes | 252 (0.1) | 168 (0.1) |
| **A stroke or TIA** | No | 348,846 (99.1) | 201,327 (98.8) |
|  | Yes | 3,169 (0.9) | 2,483 (1.2) |
| **A thrombosis or pulmonary embolus** | No | 348,904 (99.1) | 201,497 (98.9) |
|  | Yes | 3,111 (0.9) | 2,313 (1.1) |
| **Housing category** | neither | 351,669 (99.9) | 203,583 (99.9) |
|  | care home | 128 (0.0) | 92 (0.0) |
|  | homeless | 218 (0.1) | 135 (0.1) |
| **Learning disability or Down’s Syndrome** | Neither | 347,870 (98.8) | 201,265 (98.8) |
|  | learning disability | 4,065 (1.2) | 2,498 (1.2) |
|  | Down’s Syndrome | 80 (0.0) | 47 (0.0) |
| **Kidney disease** | 0 | 348,645 (99.0) | 201,284 (98.8) |
|  | 3 | 3,008 (0.9) | 2,335 (1.1) |
|  | 4 | 146 (0.0) | 82 (0.0) |
|  | 5 | 216 (0.1) | 109 (0.1) |
| **Urban-Rural Index (2016)** | 1 Large Urban Areas | 118,582 (33.7) | 69,144 (33.9) |
|  | 2 Other Urban Areas | 146,604 (41.6) | 81,017 (39.8) |
|  | 3 Accessible Small Towns | 31,593 (9.0) | 19,057 (9.4) |
|  | 4 Remote Small Towns | 13,159 (3.7) | 8,552 (4.2) |
|  | 5 Accessible Rural | 29,720 (8.4) | 17,599 (8.6) |
|  | 6 Remote Rural | 12,357 (3.5) | 8,441 (4.1) |
| **BMI (kg/m2)** | <20 | 11,831 (3.4) | 6,955 (3.4) |
|  | 20-24 | 45,310 (12.9) | 27,112 (13.3) |
|  | 25-29 | 246,200 (69.9) | 137,564 (67.5) |
|  | 30-34 | 27,842 (7.9) | 18,019 (8.8) |
|  | 35-39 | 12,994 (3.7) | 8,761 (4.3) |
|  | 40+ | 7,838 (2.2) | 5,399 (2.6) |
| **Was the person advised to shield** | No | 342,208 (97.2) | 196,636 (96.5) |
|  | Yes | 9,807 (2.8) | 7,174 (3.5) |
| **Was the person in an immuno- compromised group** | No | 342,344 (97.3) | 197,521 (96.9) |
|  | Yes | 9,671 (2.7) | 6,289 (3.1) |
| **Smoking** | Ex-Smoker | 43,346 (12.3) | 26,287 (12.9) |
|  | Non-Smoker | 170,759 (48.5) | 98,319 (48.2) |
|  | Smoker | 80,445 (22.9) | 47,331 (23.2) |
|  | Unknown | 57,465 (16.3) | 31,873 (15.6) |
| **Blood Pressure** | High | 28,798 (8.2) | 17,783 (8.7) |
|  | Low | 6,791 (1.9) | 3,780 (1.9) |
|  | No Investigation | 80,314 (22.8) | 43,422 (21.3) |
|  | Normal | 230,891 (65.6) | 135,449 (66.5) |
|  | Very High | 5,221 (1.5) | 3,376 (1.7) |

# Table B: Conditions of QCovid risk algorithm

| **QCovid risk group** |
| --- |
| Atrial fibrillation |
| Asthma |
| Blood cancer |
| Heart failure |
| Cerebral palsy |
| Coronary heart disease |
| Cirrhosis |
| Congenital heart disease |
| COPD |
| Dementia |
| Diabetes type 1 |
| Diabetes type 2 |
| Epilepsy |
| Fracture |
| Neurological disorder |
| Parkinson’s |
| Pulmonary hypertension |
| Pulmonary rare |
| Peripheral vascular disease |
| Rheumatoid arthritis or SLE |
| Respiratory cancer |
| Severe mental illness |
| Sickle cell disease |
| Stroke/TIA |
| Thrombosis or pulmonary embolus |
| Care housing category |
| Learning disability or Down's |
| Kidney disease |
| More information on codes: https://github.com/EAVE-II/EAVE-II-data-dictionary  Ref: Clift, A.K., et al. Living risk prediction algorithm (QCovid) for risk of hospital admission and mortality  from coronavirus 19 in adults: national derivation and validation cohort study. BMJ 371, m3731 (2020) [1] |

# Table C. Vaccination Status of individuals tested for SARS-CoV-2 in Brazil (A) and Scotland (B), according to the test result and severity of disease. Vaccinees data was detailed according to the time after each dose.

1. Brazil

| **Characteristic - n (%)** | **Controls** | **Symptomatic Infection** | **Severe Outcomes** |
| --- | --- | --- | --- |
| **Vaccination Status** |  |  |  |
| **Unvaccinated** | 126,679 (38.3) | 193,608 (58.6) | 10,356 (3.1) |
| *Primary series with ChAdOx1* | | | |
| **First dose** |  |  |  |
| 0 – 13 days | 430 (61.7) | 263 (37.7) | 4 (0.6) |
| 14 – 59 days | 1,343 (51.5) | 1,248 (47.9) | 17 (0.7) |
| 2 – 5 months | 18,930 (42.0) | 25,931 (57.6) | 187 (0.4) |
| ≥ 6 months | 41,120 (39.7) | 60,813 (58.7) | 1,606 (1.6) |
| **Second dose** |  |  |  |
| 0 – 13 | 2,686 (48.7) | 2,805 (50.9) | 24 (0.4) |
| 14 – 59 days | 19,434 (45.4) | 23,179 (54.2) | 156 (0.4) |
| 2 – 4 months | 407,927 (34.8) | 759,520 (64.8) | 4,487 (0.4) |
| ≥ 5 months | 266,484 (39.2) | 401,275 (59.0) | 12,105 (1.8) |
| **Booster dose (BNT162b2)** |  |  |  |
| 0 – 13 days | 74,672 (45.3) | 89,521 (54.3) | 742 (0.4) |
| 14 – 29 days | 100,448 (51.6) | 93,654 (48.1) | 719 (0.4) |
| 1 month | 146,125 (48.6) | 152,394 (50.7) | 2,024 (0.7) |
| 2 months | 94,667 (50.7) | 89,821 (48.1) | 2,063 (1.1) |
| 3 months | 43,083 (55.2) | 34,116 (43.7) | 863 (1.1) |
| ≥ 4 months | 14,374 (68.1) | 6,403 (30.3) | 324 (1.5) |
| *Primary series with BNT162b2* | | | |
| **First dose** |  |  |  |
| 0 – 13 days | 2,290 (50.8) | 2,174 (48.2) | 47 (1.0) |
| 14 – 59 days | 9,213 (51.9) | 8,350 (47.0) | 194 (1.1) |
| 2 – 5 months | 71,985 (46.2) | 82,971 (53.3) | 690 (0.4) |
| ≥ 6 months | 28,511 (51.8) | 26,188 (47.6) | 335 (0.6) |
| **Second dose** |  |  |  |
| 0 – 13 | 9,067 (60.0) | 6,011 (39.7) | 46 (0.3) |
| 14 – 59 days | 85,901 (56.9) | 64,872 (42.9) | 305 (0.2) |
| 2 – 4 months | 553,183 (43.2) | 725,973 (56.7) | 2,341 (0.2) |
| ≥ 5 months | 63,528 (55.2) | 50,919 (44.3) | 540 (0.5) |
| **Booster dose (BNT162b2)** |  |  |  |
| 0 – 13 days | 22,886 (47.9) | 24,779 (51.9) | 86 (0.2) |
| 14 – 29 days | 23,889 (52.8) | 21,285 (47.0) | 104 (0.2) |
| 1 month | 26,315 (58.9) | 18,238 (40.8) | 111 (0.2) |
| 2 months | 11,191 (73.9) | 3,925 (25.9) | 35 (0.2) |
| 3 months | 2,971 (76.4) | 910 (23.4) | 8 (0.2) |
| ≥ 4 months | 442 (75.0) | 144 (24.4) | 3 (0.5) |

The percentages were calculated using the sum of individuals in each row as the denominator.

1. Scotland

| **Characteristic – n (%)** | **Controls** | **Symptomatic Infection** | **Severe Outcomes** | **Confirmed Severe Outcomes** |
| --- | --- | --- | --- | --- |
| **Vaccination Status** |  |  |  |  |
| **Unvaccinated** | 13,250 (25.2) | 38,988 (74.3) | 169 (0.3) | 89 (0.2) |
| *Primary series with ChAdOx1* | | | | |
| **First dose** | 1,026 (30.5) | 2,307 (68.5) | 23 (0.7) | 11 (0.3) |
| **Second dose** |  |  |  |  |
| 0 – 4 months | 1,264 (32.2) | 2,641 (67.4) | 10 (0.3) | 5 (0.1) |
| ≥ 5 months | 9,543 (27.3) | 25,267 (72.2) | 130 (0.4) | 62 (0.2) |
| **Booster dose (BNT162b2)** |  |  |  |  |
| 0 – 13 days | 3,783 (40.7) | 5,498 (59.1) | 11 (0.1) | 6 (0.1) |
| 14 – 29 days | 7,793 (50.1) | 7,725 (49.7) | 23 (0.1) | 7 (0) |
| 1 month | 13,859 (47.6) | 15,182 (52.1) | 68 (0.2) | 29 (0.1) |
| 2 months | 10,849 (39) | 16,790 (60.4) | 120 (0.4) | 62 (0.2) |
| 3 months | 8,880 (33.3) | 17,636 (66.2) | 88 (0.3) | 40 (0.2) |
| ≥ 4 months | 5,179 (31.1) | 11,385 (68.3) | 71 (0.4) | 35 (0.2) |
| **Booster dose (mRNA-1273)** |  |  |  |  |
| 0 – 13 days | 1,799 (41.7) | 2,508 (58.2) | 1 (0) | 1 (0) |
| 14 – 29 days | 3,458 (54.3) | 2,906 (45.6) | 5 (0.1) | 1 (0) |
| 1 month | 5,707 (48.5) | 6,036 (51.3) | 20 (0.2) | 9 (0.1) |
| 2 months | 4,199 (39.1) | 6,496 (60.6) | 21 (0.2) | 10 (0.1) |
| 3 months | 3,636 (34.4) | 6,905 (65.3) | 20 (0.2) | 9 (0.1) |
| ≥ 4 months | 1,866 (33.2) | 3,720 (66.3) | 18 (0.3) | 9 (0.2) |
| **Second Booster (mRNA-1273 or BNT162b2)** | 679 (36.6) | 1,148 (61.9) | 21 (1.1) | 7 (0.4) |
| *Primary series with BNT162b2* | | | | |
| **First dose** | 3,819 (31.1) | 8,436 (68.6) | 28 (0.2) | 11 (0.1) |
| **Second dose** |  |  |  |  |
| 0 – 4 months | 14,536 (32.3) | 30,374 (67.6) | 44 (0.1) | 11 (0) |
| ≥ 5 months | 12,545 (27.9) | 32,274 (71.8) | 77 (0.2) | 27 (0.1) |
| **Booster dose (BNT162b2)** |  |  |  |  |
| 0 – 13 days | 5,897 (42) | 8,143 (57.9) | 9 (0.1) | 5 (0) |
| 14 – 29 days | 6,002 (52.3) | 5,463 (47.6) | 10 (0.1) | 3 (0) |
| 1 month | 11,418 (46.6) | 13,053 (53.2) | 40 (0.2) | 16 (0.1) |
| 2 months | 13,511 (40.1) | 20,109 (59.6) | 70 (0.2) | 30 (0.1) |
| 3 months | 9,678 (38.2) | 15,564 (61.5) | 49 (0.2) | 18 (0.1) |
| ≥ 4 months | 9,370 (33.2) | 18,714 (66.4) | 83 (0.3) | 33 (0.1) |
| **Booster dose (mRNA-1273)** |  |  |  |  |
| 0 – 13 days | 2,860 (44.5) | 3,564 (55.4) | 8 (0.1) | (0) |
| 14 – 29 days | 2,882 (55.4) | 2,315 (44.5) | 4 (0.1) | 1 (0) |
| 1 month | 4,907 (47.9) | 5,326 (52) | 13 (0.1) | 4 (0) |
| 2 months | 4,410 (40) | 6,594 (59.8) | 15 (0.1) | 3 (0) |
| 3 months | 2,951 (37.9) | 4,814 (61.9) | 10 (0.1) | 2 (0) |
| ≥ 4 months | 1,498 (34.1) | 2,890 (65.7) | 7 (0.2) | 2 (0) |
| **Second Booster (mRNA-1273 or BNT162b2)** | 330 (35.5) | 574 (61.8) | 15 (1.6) | 10 (1.1) |

The percentages were calculated using the sum of individuals in each row as the denominator.

# Table D. Time interval in days - median (Interquartile Interval) - between vaccination and test in Brazil and Scotland, according to the type of vaccine used in the primary series.

|  | **Brazil** | **Scotland** |
| --- | --- | --- |
| ***Primary series with ChAdOx1*** | | |
| **First dose** |  |  |
| 0 – 13 days | 0 (5 to 9) | 11 (7 to 12) |
| 14 – 59 days | 31 (42 to 51) | 44 (35 to 51) |
| 2 – 5 months | 144 (160 to 171) | 141 (114 to 164) |
| ≥ 6 months | 198 (218 to 246) | 295 (262 to 329) |
| **Second dose** |  |  |
| 0 – 13 | 4 (7 to 10) | 8 (6 to 9) |
| 14 – 59 days | 36 (48 to 55) | 41 (30 to 51) |
| 2 – 4 months | 101 (119 to 133) | 127 (103 to 141) |
| ≥ 5 months | 161 (174 to 196) | 214 (188 to 244) |
| **Booster dose (BNT162b2)** |  |  |
| 0 – 13 days | 4 (7 to 10) | 8 (5 to 11) |
| 14 – 29 days | 17 (22 to 26) | 22 (18 to 26) |
| 1 month | 36 (43 to 50) | 44 (37 to 51) |
| 2 months | 66 (72 to 80) | 75 (67 to 83) |
| 3 months | 94 (100 to 108) | 103 (96 to 111) |
| ≥ 4 months | 125 (132 to 144) | 134 (126 to 146) |
| **Booster dose (mRNA-1273)** |  |  |
| 0 – 13 days | – | 7 (4 to 10) |
| 14 – 29 days | – | 22 (18 to 26) |
| 1 month | – | 43 (36 to 51) |
| 2 months | – | 75 (68 to 82) |
| 3 months | – | 103 (96 to 111) |
| ≥ 4 months | – | 132 (125 to 142) |
| **Second booster dose (BNT162b2)** |  |  |
| 0 – 6 days | – | 3 (2 to 5) |
| 7 – 29 days | – | 17 (11 to 22) |
| ≥ 1 month | – | 60 (47 to 72) |
| **Second booster dose (mRNA-1273)** |  |  |
| 0 – 6 days | – | 4 (2 to 5) |
| 7 – 29 days | – | 15 (10 to 23) |
| ≥ 1 month | – | 60 (48 to 75) |
| ***Primary series with BNT162b2*** | | |
| **First dose** |  |  |
| 0 – 13 days | 3 (7 to 10) | 8 (4 to 10) |
| 14 – 59 days | 28 (41 to 50) | 39 (27 to 50) |
| 2 – 5 months | 125 (148 to 161) | 117 (89 to 154) |
| ≥ 6 months | 189 (201 to 217) | 230 (202 to 269) |
| **Second dose** |  |  |
| 0 – 13 | 3 (6 to 9) | 7 (4 to 10) |
| 14 – 59 days | 38 (49 to 55) | 42 (30 to 51) |
| 2 – 4 months | 83 (101 to 119) | 127 (109 to 138) |
| ≥ 5 months | 156 (164 to 176) | 197 (170 to 252) |
| **Booster dose (BNT162b2)** |  |  |
| 0 – 13 days | 4 (7 to 10) | 7 (4 to 10) |
| 14 – 29 days | 17 (21 to 25) | 20 (17 to 25) |
| 1 month | 35 (41 to 49) | 46 (38 to 53) |
| 2 months | 64 (70 to 78) | 76 (68 to 83) |
| 3 months | 93 (99 to 106) | 101 (95 to 110) |
| ≥ 4 months | 123 (128 to 135) | 147 (132 to 164) |
| **Booster dose (mRNA-1273)** |  |  |
| 0 – 13 days | – | 6 (4 to 10) |
| 14 – 29 days | – | 21 (17 to 25) |
| 1 month | – | 46 (38 to 53) |
| 2 months | – | 75 (68 to 82) |
| 3 months | – | 101 (95 to 109) |
| ≥ 4 months | – | 139 (127 to 155) |
| **Second booster dose (BNT162b2)** |  |  |
| 0 – 6 days | – | 3 (2 to 5) |
| 7 – 29 days | – | 17 (12 to 24) |
| ≥ 1 month | – | 62 (48 to 74) |
| **Second booster dose (mRNA-1273)** |  |  |
| 0 – 6 days | – | 3 (2 to 5) |
| 7 – 29 days | – | 14 (10 to 21) |
| ≥ 1 month | – | 58 (44 to 69) |

# Table E. Vaccine Effectiveness against symptomatic infection in Brazil and Scotland, expressed in percentages (95% CI), according to the type of vaccine used at the primary series and by age group. Reference group: individuals unvaccinated.

1. **Brazil**
2. Primary series with ChAdOx1

| **Period** | **Overall** | **18-49 years** | **50-64 years** | **≥ 65 years** |
| --- | --- | --- | --- | --- |
| **First dose** |  |  |  |  |
| 0 – 13 days | 59.4 (52.5 to 65.3)** | 55.8 (47.8 to 62.6)** | 75.2 (57.6 to 85.5)** | 60.1 (−7.2 to 85.2)† |
| 14 – 59 days | 33 (27.4 to 38.1)** | 30.5 (24.3 to 36.2)** | 36.3 (18.7 to 50.1)** | 48.3 (10.6 to 70.2)* |
| 2 – 5 months | 19.2 (17.5 to 20.8)** | 16.1 (14.3 to 17.9)** | 33.1 (26.5 to 39.1)** | 41.6 (22.4 to 56.0)** |
| ≥ 6 months | 8.1 (6.7 to 9.5)** | 3.7 (2.0 to 5.4)** | 17.9 (15.1 to 20.5)** | 23.5 (18.1 to 28.5)** |
| **Second dose** |  |  |  |  |
| 0 – 13 days | 39.1 (35.7 to 42.4)** | 36.7 (32.8 to 40.3)** | 44.1 (34.7 to 52.1)** | 52.8 (21.1 to 71.7)* |
| 14 – 59 days | 26.7 (25.2 to 28.2)** | 23.4 (21.7 to 25.1)** | 42.9 (38.5 to 46.9)** | 55.0 (43.0 to 64.5)** |
| 2 – 4 months | −0.3 (−1.2 to 0.6)† | −5.8 (−6.8 to −4.7)** | 15.2 (13.4 to 17.0)** | 34.9 (30.1 to 39.4)** |
| ≥ 5 months | 0.9 (0.0 to 1.9)† | −5.1 (−6.4 to −3.9)** | 12.7 (10.8 to 14.4)** | 21.1 (18.5 to 23.7)** |
| **Booster dose (BNT162b2)** |  |  |  |  |
| 0 – 13 days | 37.6 (36.8 to 38.4)** | 35.4 (34.3 to 36.5) | 42.2 (40.7 to 43.6)** | 45.6 (41.9 to 49.1)** |
| 14 – 29 days | 51.6 (51.0 to 52.2)** | 47.4 (46.5 to 48.2) | 56.4 (55.3 to 57.4)** | 62.2 (60.2 to 64.1)** |
| 1 month | 38.1 (37.4 to 38.8)** | 35.1 (34.1 to 36.2) | 41.5 (40.1 to 42.8)** | 49.0 (47.1 to 50.9)** |
| 2 months | 21.5 (20.4 to 22.7)** | 17.5 (15.7 to 19.2) | 29.8 (27.8 to 31.6)** | 31.4 (28.9 to 33.9)** |
| 3 months | 12.6 (10.8 to 14.4)** | 3.2 (0.3 to 6.0) | 26.1 (23.1 to 28.9)** | 26.6 (23.1 to 29.9)** |
| ≥ 4 months | 4.2 (0.7 to 7.6)* | −11.8 (−18.0 to −6.0) | 12.3 (5.9 to 18.3)** | 30.5 (25.3 to 35.4)** |

†p>0.05; *p≤0.05, **p<0.001 – p-values for Wald’s test two-sided.

1. Primary series with BNT162b2

| **Period** | **Overall** | **18-49 years** | **50-64 years** |
| --- | --- | --- | --- |
| **First dose** |  |  |  |
| 0 – 13 days | 45.2 (41.8 to 48.4)** | 43.9 (40.2 to 47.4)** | 48.5 (37.2 to 57.8)** |
| 14 – 59 days | 42.6 (40.8 to 44.4)** | 42.1 (40.1 to 44.0)** | 40.8 (33.6 to 47.2)** |
| 2 – 5 months | 28.3 (27.4 to 29.3)** | 26.9 (25.9 to 27.9)** | 31.9 (27.7 to 36.0)** |
| ≥ 6 months | 22.1 (20.6 to 23.7)** | 21.0 (19.3 to 22.7)** | 22.9 (18.4 to 27.2)** |
| **Second dose** |  |  |  |
| 0 – 13 days | 59.1 (57.6 to 60.5)** | 58.5 (57.0 to 60.0)** | 58.8 (52.7 to 64.1)** |
| 14 – 59 days | 49.2 (48.5 to 49.9)** | 48.2 (47.5 to 48.9)** | 57.4 (54.6 to 60.1)** |
| 2 – 4 months | 19.4 (18.7 to 20.1)** | 17.3 (16.6 to 18.1)** | 29.7 (27.9 to 31.4)** |
| ≥ 5 months | 9.6 (8.1 to 11.0)** | 4.9 (3.1 to 6.6)** | 22.5 (19.9 to 25.0)** |
| **Booster dose (BNT162b2)** |  |  |  |
| 0 – 13 days | 41.3 (40.0 to 42.5)** | 40.3 (38.9 to 41.8)** | 43.8 (41.2 to 46.3)** |
| 14 – 29 days | 44.6 (43.4 to 45.8)** | 42.3 (40.7 to 43.8)** | 49.0 (46.7 to 51.2)** |
| 1 month | 34.6 (33.0 to 36.2)** | 30.9 (28.8 to 32.9)** | 39.9 (37.1 to 42.6)** |
| 2 months | 23.4 (19.9 to 26.7)** | 13.0 (8.3 to 17.5)** | 41.4 (36.3 to 46.0)** |
| 3 months | 2.8 (−5.5 to 10.5)† | −10.0 (−21.9 to 0.7)† | 26.2 (14.8 to 36.1)** |
| ≥ 4 months | −11.8 (−35.9 to 8.0)† | −23.5 (−64.2 to 7.2)† | - 1. (−19.6 to 31.1)† |

†p>0.05; *p≤0.05, **p<0.001 – p-values for Wald’s test two-sided.

1. **Scotland**
2. Primary series with ChAdOx1

| **Period** | **Overall** | **18-49 years** | **50-64 years** | **≥ 65 years** |
| --- | --- | --- | --- | --- |
| **First dose** |  |  |  |  |
| 0 – 13 days | 81.3 (-116.9 to 98.4)† | § | § | § |
| 14 – 59 days | 55.4 (4.5 to 79.2) | § | § | § |
| 2 – 5 months | 18.5 (-11.4 to 40.4)† | 17.5 (-17.2 to 41.9)† | § | § |
| ≥ 6 months | 22.8 (15.4 to 29.5) | 22.3 (13.6 to 30.2)** | 20.5 (3.9 to 34.2)* | 32.9 (-39.9 to 67.8)† |
| **Second dose** |  |  |  |  |
| 0 – 13 days | 42.2 (-2.5 to 67.4)† | 7.4 (-98.4 to 56.8)† | § | § |
| 14 – 59 days | 40.7 (26.6 to 52.1)** | 38.1 (21.0 to 51.5)** | 47.7 (17.8 to 66.7)* | § |
| 2 – 4 months | 23.9 (17.1 to 30.2)** | 24.1 (16.5 to 30.9)** | 29.3 (12.8 to 42.7)* | § |
| ≥ 5 months | 9.6 (6.3 to 12.9)** | 8.9 (5.1 to 12.6)** | 6.1 (-3.2 to 14.6)† | 8.6 (-24.6 to 33)† |
| **Booster dose (BNT162b2)** |  |  |  |  |
| 0 – 13 days | 50.2 (47.4 to 52.8)** | 50.9 (47.4 to 54.1)** | 43.7 (37.0 to 49.6)** | 58.2 (26.4 to 76.3)* |
| 14 – 29 days | 67.1 (65.5 to 68.5)** | 66.6 (64.5 to 68.5)** | 63.8 (60.0 to 67.1)** | 59.1 (41.9 to 71.2)** |
| 1 month | 62.1 (60.6 to 63.5)** | 63.0 (61.0 to 64.8)** | 57.7 (53.6 to 61.3)** | 61.5 (50.5 to 70.1)** |
| 2 months | 53.5 (51.6 to 55.4)** | 55.5 (53.0 to 57.9)** | 52.0 (47.2 to 56.3)** | 45.1 (30.1 to 56.8)** |
| 3 months | 47.4 (45.0 to 49.7)** | 50.5 (47.4 to 53.4)** | 44.9 (39.2 to 49.9)** | 46.7 (32.0 to 58.3)** |
| ≥ 4 months | 37.4 (33.8 to 40.9)** | 39.4 (33.5 to 44.7)** | 35.0 (27.4 to 41.9)** | 36.7 (18.6 to 50.8)** |
| **Booster dose (mRNA-1273)** |  |  |  |  |
| 0 – 13 days | 52.0 (48.4 to 55.4)** | 52.8 (48.2 to 57.0)** | 46.8 (39.2 to 53.5)** | 38.6 (-22.1 to 69.1)† |
| 14 – 29 days | 71.7 (69.9 to 73.3)** | 72.9 (70.4 to 75.1)** | 67.1 (63.2 to 70.6)** | 76.0 (62.9 to 84.4)** |
| 1 month | 63.7 (61.9 to 65.5)** | 62.8 (60.3 to 65.3)** | 62.1 (58.0 to 65.8)** | 61.0 (48.8 to 70.2)** |
| 2 months | 56.6 (54.3 to 58.9)** | 59.3 (56.3 to 62.0)** | 53.7 (48.4 to 58.5)** | 49.6 (34.1 to 61.4)** |
| 3 months | 52.8 (50.1 to 55.4)** | 56.1 (52.5 to 59.4)** | 51.4 (45.8 to 56.4)** | 46.0 (28.5 to 59.3)** |
| ≥ 4 months | 44.1 (39.7 to 48.1)** | 45.0 (37.0 to 51.9)** | 47.0 (39.6 to 53.5)** | 31.3 (9.2 to 48.1)** |
| **2nd Booster dose (BNT162b2)** |  |  |  |  |
| 0 – 6 days | 29.7 (-18.3 to 58.2)† | 23.3 (-92.5 to 69.5)† | -18.4 (-236.6 to 58.3)† | 50.8 (-16.8 to 79.3)† |
| 7 – 29 days | 40.6 (20.9 to 55.4)** | 43.9 (10.2 to 65.0)* | 42.4 (7.8 to 64.1)* | 29.1 (-31.3 to 61.7)† |
| ≥ 1 month | 46.9 (36.6 to 55.6)** | 50.7 (33.1 to 63.7)** | 40.9 (20.6 to 56)** | 51.4 (26.5 to 67.8)** |
| **2nd Booster dose (mRNA-1273)** |  |  |  |  |
| 0 – 6 days | 43.6 (-1.7 to 68.7)† | 62.0 (-4.1 to 86.2)† | 24.8 (-155.7 to 77.9)† | 28.9 (-84.5 to 72.6)† |
| 7 – 29 days | 47.5 (21.5 to 64.9)** | 59.4 (25.6 to 77.8)* | 21.1 (-80.9 to 65.6)† | 40.3 (-27.9 to 72.2)† |
| ≥ 1 month | 54.1 (40.6 to 64.6)** | 65.3 (48.0 to 76.8)** | 54.8 (30.6 to 70.6)** | 25.8 (-35.2 to 59.3)† |

†p>0.05; *p≤0.05; **p<0.001 – p-values for Wald’s test two-sided.

§ indicates a period without enough cases and controls to reliably estimate the vaccine effectiveness

1. Primary series with BNT162b2

| **Period** | **Overall** | **18-49 years** | **50-64 years** | **≥ 65 years** |
| --- | --- | --- | --- | --- |
| **First dose** |  |  |  |  |
| 0 – 13 days | 31.1 (12.0 to 46.0)* | 34.3 (15.7 to 48.7)** | § | § |
| 14 – 59 days | 40.6 (33.6 to 46.9)** | 40.1 (32.9 to 46.5)** | 70.7 (46.0 to 84.1)** | § |
| 2 – 5 months | 19.4 (12.9 to 25.5)** | 20.2 (13.6 to 26.2)** | 24.1 (-41.3 to 59.3)† | § |
| ≥ 6 months | 23.2 (17.9 to 28.2)** | 22.6 (17.2 to 27.7)** | 37.6 (1.4 to 60.5)* | § |
| **Second dose** |  |  |  |  |
| 0 – 13 days | 60.7 (53.4 to 66.8)** | 61.6 (54.5 to 67.6)** | § | § |
| 14 – 59 days | 57.7 (54.5 to 60.6)** | 58.5 (55.4 to 61.3)** | 43.4 (-3.7 to 69.1)† | § |
| 2 – 4 months | 21.3 (18.6 to 23.9)** | 23.4 (20.7 to 26.1)** | 20.3 (-20 to 47.1)† | § |
| ≥ 5 months | 17.8 (15.1 to 20.5)** | 18.6 (15.7 to 21.3)** | -0.9 (-15.5 to 11.8)† | 17.8 (-14.8 to 41.1)† |
| **Booster dose (BNT162b2)** |  |  |  |  |
| 0 – 13 days | 54.1 (52.0 to 56.1)** | 55.1 (53.0 to 57.1)** | 54.1 (43.2 to 62.9)** | 66.0 (36.1 to 81.9)** |
| 14 – 29 days | 69.1 (67.6 to 70.5)** | 68.9 (67.3 to 70.5)** | 66.9 (61.1 to 71.8)** | 68.5 (53.6 to 78.6)** |
| 1 month | 63.0 (61.6 to 64.3)** | 61.7 (60.0 to 63.3)** | 62.1 (57.3 to 66.3)** | 53.4 (40.1 to 63.7)** |
| 2 months | 56.9 (55.4 to 58.4)** | 55.6 (53.8 to 57.4)** | 57.3 (52.5 to 61.6)** | 43.7 (28.3 to 55.7)** |
| 3 months | 53.6 (51.7 to 55.4)** | 54.4 (52.2 to 56.5)** | 54.2 (49.1 to 58.8)** | 35.0 (16.9 to 49.2)** |
| ≥ 4 months | 49.3 (47.0 to 51.5)** | 55.1 (52.6 to 57.5)** | 48.1 (42.0 to 53.5)** | 29.5 (9.5 to 45.1)* |
| **Booster dose**  **(mRNA-1273)** |  |  |  |  |
| 0 – 13 days | 57.9 (55.3 to 60.4)** | 59.5 (56.9 to 62.0)** | 43.1 (27.7 to 55.2)** | 32.3 (-55.6 to 70.5)† |
| 14 – 29 days | 71.9 (70.0 to 73.7)** | 72.6 (70.6 to 74.5)** | 61.4 (52.5 to 68.6)** | 75.4 (58.3 to 85.5)** |
| 1 month | 64.8 (62.9 to 66.5)** | 63.4 (61.3 to 65.5)** | 65.6 (59.5 to 70.8)** | 57.4 (44.4 to 67.3)** |
| 2 months | 59.4 (57.3 to 61.4)** | 60.0 (57.7 to 62.2)** | 50.0 (41.5 to 57.3)** | 50.7 (35.6 to 62.3)** |
| 3 months | 55.6 (52.9 to 58.2)** | 57.7 (54.7 to 60.5)** | 51.9 (43.4 to 59.1)** | 40.0 (20.9 to 54.5)** |
| ≥ 4 months | 49.3 (45.1 to 53.2)** | 54.2 (48.2 to 59.4)** | 43.5 (32.1 to 53.1)** | 41.8 (23.9 to 55.5)** |
| **2nd Booster dose (BNT162b2)** |  |  |  |  |
| 0 – 6 days | 33.7 (-28.4 to 65.8)** | 60.4 (-65.4 to 90.5)† | 38.2 (-88.9 to 79.8)† | -14.6 (-239 to 61.3)† |
| 7 – 29 days | 61.1 (43.6 to 73.2)** | 64.6 (40.1 to 79.1)** | 65.3 (28.8 to 83.1)* | 44.0 (-29.5 to 75.8)† |
| ≥ 1 month | 49.0 (34.3 to 60.4)** | 45.2 (17.7 to 63.6)* | 60.8 (40.8 to 74.0)** | 33.0 (-21.8 to 63.2)† |
| **2nd Booster dose (mRNA-1273)** |  |  |  |  |
| 0 – 6 days | 60.7 (10.5 to 82.7)* | 51.3 (-75.3 to 86.5)† | § | 34.6 (-135.3 to 81.8)† |
| 7 – 29 days | 69.2 (46.5 to 82.3)** | 53.2 (-22.7 to 82.2)† | 62.1 (-12.9 to 87.3)† | 79.8 (48.1 to 92.1)** |
| ≥ 1 month | 55.1 (33.3 to 69.7)** | 51.2 (6.3 to 74.5)* | 68.4 (36.2 to 84.3)* | 40.3 (-28.3 to 72.2)† |

†p>0.05; *p≤0.05; **p<0.001– p-values for Wald’s test two-sided.

§ indicates a period without enough cases and controls to reliably estimate the vaccine effectiveness

# Table F. Vaccine Effectiveness against severe outcomes in Brazil and Scotland, expressed in percentages (95% CI), according to the type of vaccine used at the primary series and by age group. Reference group: individuals unvaccinated

1. **Brazil**
2. Primary series with ChAdOx1

| **Period** | **Overall** | **18-49 years** | **50-64 years** | **≥ 65 years** |
| --- | --- | --- | --- | --- |
| **First dose** |  |  |  |  |
| 0 – 13 days | 75.7 (28.8 to 91.7)** | 67.3 (-13.9 to 90.6)† | § | 77.9 (-140.1 to 98.0)† |
| 14 – 59 days | 67.0 (44.2 to 80.5)** | 73.3 (38.2 to 88.4)* | 56.4 (-12.4 to 83.1)† | 64.1 (4.2 to 86.5)* |
| 2 – 5 months | 64.1 (58.1 to 69.2)** | 69.2 (63.0 to 74.4)** | 53.2 (34.2 to 66.7)** | 58.2 (26.8 to 76.1)* |
| ≥ 6 months | 51.5 (48.3 to 54.6)** | 52.9 (47.4 to 57.8)** | 60.8 (56.2 to 65.0)** | 38.4 (30.9 to 45.0)** |
| **Second dose** |  |  |  |  |
| 0 – 13 days | 79.7 (68.9 to 86.7)** | 82.9 (68.2 to 90.8)** | 78.9 (56.9 to 89.7)** | 70.9 (10.4 to 90.6)* |
| 14 – 59 days | 77.1 (72.9 to 80.6)** | 82.5 (77.8 to 86.2)** | 72.5 (62.9 to 79.6)** | 69 (50.4 to 80.6)** |
| 2 – 4 months | 76.3 (75.2 to 77.3)** | 68.2 (65.7 to 70.6)** | 79.4 (77.9 to 80.9)** | 61.1 (54.9 to 66.5)** |
| ≥ 5 months | 67.8 (66.7 to 69.0)** | 78 (76.5 to 79.4)** | 74.6 (72.9 to 76.1)** | 58.3 (55.9 to 60.6)** |
| **Booster dose (BNT162b2)** |  |  |  |  |
| 0 – 13 days | 88.1 (87.1 to 89.1)** | 91.6 (90.0 to 93.0)** | 90.4 (89.1 to 91.5)** | 77.4 (73.3 to 80.8) |
| 14 – 29 days | 93.5 (93.0 to 94.0)** | 94.3 (93.1 to 95.3)** | 94.9 (94.2 to 95.5)** | 90.1 (88.5 to 91.5) |
| 1 month | 91.9 (91.4 to 92.3)** | 93.5 (92.2 to 94.5)** | 94.2 (93.6 to 94.8)** | 88.2 (87.2 to 89.2) |
| 2 months | 88.3 (87.5 to 89.0)** | 90.3 (87.6 to 92.4)** | 93.7 (92.7 to 94.6)** | 84.9 (83.7 to 86.0) |
| 3 months | 86.2 (84.9 to 87.4)** | 87.9 (83.0 to 91.4)** | 91.9 (89.6 to 93.7)** | 83.7 (81.9 to 85.2) |
| ≥ 4 months | 82.3 (79.7 to 84.7)** | 82.3 (67.2 to 90.4)** | 85.8 (77.4 to 91.1)** | 80.2 (76.9 to 83.0) |

†p>0.05; *p≤0.05; **p<0.001– p-values for Wald’s test two-sided.

§ indicates a period without enough cases and controls to reliably estimate the vaccine effectiveness

1. Primary series with BNT162b2

| **Period** | **Overall** | **18-49 years** | **50-64 years** |
| --- | --- | --- | --- |
| **First dose** |  |  |  |
| 0 – 13 days | 60.2 (42.3 to 72.5)** | 72.1 (52.3 to 83.7)** | 32.5 (−15.9 to 60.7)† |
| 14 – 59 days | 45.4 (34.7 to 54.3)** | 45.0 (32.2 to 55.3)** | 47.1 (24.8 to 62.8)** |
| 2 – 5 months | 63.1 (59.5 to 66.3)** | 65.1 (61.3 to 68.6)** | 48.2 (36.0 to 58.2)** |
| ≥ 6 months | 63.7 (58.8 to 68.0)** | 65.1 (59.3 to 70.1)** | 60.2 (50.3 to 68.1)** |
| **Second dose** |  |  |  |
| 0 – 13 days | 83.0 (76.3 to 87.8)** | 81.4 (73.5 to 87.0)** | 88.6 (71.8 to 95.4)** |
| 14 – 59 days | 82.9 (80.6 to 85.0)** | 83.8 (81.3 to 85.9)** | 79.3 (71.8 to 84.9)** |
| 2 – 4 months | 84.9 (84.0 to 85.7)** | 84.7 (83.6 to 85.7)** | 86.1 (84.5 to 87.6)** |
| ≥ 5 months | 82.6 (80.7 to 84.3)** | 81.8 (78.9 to 84.4)** | 82.1 (79.3 to 84.6)** |
| **Booster dose (BNT162b2)** |  |  |  |
| 0 – 13 days | 92.8 (91.0 to 94.2)** | 91.2 (88.4 to 93.3)** | 94.6 (92.2 to 96.3)** |
| 14 – 29 days | 92.7 (91.0 to 94.0)** | 90.9 (88.1 to 93.1)** | 94.4 (92.3 to 95.9)** |
| 1 month | 92.8 (91.1 to 94.1)** | 91.4 (88.3 to 93.6)** | 93.7 (91.6 to 95.2)** |
| 2 months | 89.0 (84.4 to 92.3)** | 87.0 (77.9 to 92.4)** | 90.2 (84.2 to 93.9)** |
| 3 months | 86.6 (72.3 to 93.5)** | 95.7 (68.4 to 99.4)* | 76.0 (46.1 to 89.3)* |
| ≥ 4 months | 74.1 (9.1 to 92.6)† | § | § |

†p>0.05; *p≤0.05; **p<0.001– p-values for Wald’s test two-sided.

§ indicates a period without enough cases and controls to reliably estimate the vaccine effectiveness

1. **Scotland**

|  | **ChAdOx1** | **BNT162b2** |
| --- | --- | --- |
| **Period** | **Overall** | **Overall** |
| **First dose** |  |  |
| 0 – 13 days | § | § |
| 14 – 59 days | § | § |
| 2 – 5 months | § | § |
| ≥ 6 months | 19.3 (-58.16 to 58.82)† | 59.1 (-12.3 to 85.1)† |
| **Second dose** |  |  |
| 0 – 13 days | § |  |
| 14 – 59 days | § | 90.4 (30.4 to 98.7)* |
| 2 – 4 months | 78.8 (31.8 to 93.4)* | 84.8 (70.1 to 92.3)** |
| ≥ 5 months | 52.8 (33.3 to 66.7)** | 72.9 (57.9 to 82.5)** |
| **Booster dose (BNT162b2)** |  |  |
| 0 – 13 days | 96.3 (73.1 to 99.5)* | 89.6 (74.1 to 95.8)** |
| 14 – 29 days | 98.3 (87.3 to 99.8)** | 95.3 (85.1 to 98.5)** |
| 1 month | 92.3 (84.3 to 96.3)** | 91.2 (84.5 to 95.0)** |
| 2 months | 89.3 (78.7 to 94.7)** | 89.7 (83.6 to 93.5)** |
| 3 months | 82.2 (62.8 to 91.5)** | 88.4 (80.1 to 93.3)** |
| ≥ 4 months | 77.8 (51.4 to 89.9)** | 78.6 (64.4 to 87.1)** |
| **Booster dose (mRNA-1273)** |  |  |
| 0 – 13 days | 90.0 (76.6 to 95.7)** | § |
| 14 – 29 days | 94.4 (87.7 to 97.5)** | 96.1 (72.1 to 99.5)** |
| 1 month | 90.4 (84.7 to 93.9)** | 93.5 (82.1 to 97.7)** |
| 2 months | 81.4 (72.4 to 87.5)** | 93.4 (78.8 to 97.9)** |
| 3 months | 78.0 (65.6 to 86.0)** | 92.1 (67.3 to 98.1)** |
| ≥ 4 months | 76.0 (59.3 to 85.9)** | 90.4 (59.2 to 97.8)** |

†p>0.05; *p≤0.05; **p<0.001– p-values for Wald’s test two-sided.

§ indicates a period without enough cases and controls to reliably estimate the vaccine effectiveness

# Table G. Relative Vaccine Effectiveness against symptomatic infection in Brazil and Scotland expressed in percentages (95% CI) according to the type of vaccine used at the primary series and by age group. Reference group: individuals that received only a primary series.

1. **Brazil**
2. Primary series with ChAdOx1

| **Period** | **Overall** | **18-49 years** | **50-64 years** | **≥ 65 years** |
| --- | --- | --- | --- | --- |
| **Booster dose (BNT162b2)** |  |  |  |  |
| 0 – 13 days | 37.2 (36.6 to 37.9)** | 39.8 (38.9 to 40.6)** | 33.5 (32.3 to 34.6)** | 29.4 (25.0 to 33.5)** |
| 14 – 29 days | 50.9 (50.4 to 51.4)** | 50.7 (50.0 to 51.4)** | 49.8 (49.1 to 50.6)** | 50.9 (48.8 to 53.0)** |
| 1 month | 37.1 (36.5 to 37.6)** | 40.1 (39.3 to 40.9)** | 33.5 (32.6 to 34.4)** | 34.9 (33.1 to 36.6)** |
| 2 months | 22.1 (21.2 to 23.0)** | 27.7 (26.4 to 29.0)** | 21.7 (20.2 to 23.2)** | 14.8 (12.3 to 17.3)** |
| 3 months | 14.6 (13.1 to 16.2)** | 16.5 (14.3 to 18.7)** | 18.3 (15.5 to 21.0)** | 10.9 (7.0 to 14.6)** |
| ≥ 4 months | 7.0 (3.8 to 10.1)** | 6.5 (1.6 to 11.1)* | 4.5 (−1.9 to 10.6)† | 17.8 (11.9 to 23.3)** |

†p>0.05; *p≤0.05; **p<0.001– p-values for Wald’s test two-sided.

1. Primary series with BNT162b2

| **Period** | **Overall** | **18-49 years** | **50-64 years** |
| --- | --- | --- | --- |
| **Booster dose (BNT162b2)** |  |  |  |
| 0 – 13 days | 34.1 (32.9 to 35.4)** | 36.2 (34.8 to 37.6)** | 26.2 (23.1 to 29.1)** |
| 14 – 29 days | 40.5 (39.3 to 41.7)** | 41.6 (40.2 to 43.0)** | 34.9 (32.2 to 37.4)** |
| 1 month | 32.8 (31.3 to 34.3)** | 34.2 (32.4 to 35.9)** | 25.8 (22.5 to 28.9)** |
| 2 months | 22.9 (19.7 to 26.1)** | 19.3 (15.2 to 23.2)** | 29.7 (23.8 to 35.2)** |
| 3 months | 8.5 (1.0 to 15.5)* | 7.2 (−2.4 to 15.9)† | 17.2 (4.7 to 28.1)* |
| ≥ 4 months | 9.7 (−9.6 to 25.6)† | 17.7 (−9.2 to 38.0)† | 8.2 (−20.8 to 30.2)† |

†p>0.05; *p≤0.05; **p<0.001– p-values for Wald’s test two-sided.

1. **Scotland**
2. Primary series with ChAdOx1

| **Period** | **Overall** | **18-49 years** | **50-64 years** | **≥ 65 years** |
| --- | --- | --- | --- | --- |
| **Booster dose (BNT162b2)** |  |  |  |  |
| 0 – 13 days | 44.0 (40.9 to 46.9)** | 44.7 (40.7 to 48.4)** | 40.2 (34.7 to 45.1)** | 55.5 (21.4 to 74.8)* |
| 14 – 29 days | 63.1 (61.4 to 64.7)** | 62.5 (60.1 to 64.7)** | 61.4 (58.6 to 64.0)** | 55.3 (37.1 to 68.3)** |
| 1 month | 57.0 (55.2 to 58.6)** | 58.3 (55.9 to 60.6)** | 53.9 (50.8 to 56.7)** | 58 (46.5 to 67.1)** |
| 2 months | 47.1 (44.7 to 49.5)** | 48.9 (45.6 to 52.0)** | 45.9 (41.6 to 49.8)** | 40.9 (25 to 53.4)** |
| 3 months | 40.7 (37.5 to 43.7)** | 42.5 (38.2 to 46.6)** | 38.2 (32.7 to 43.2)** | 42.1 (25.2 to 55.2)** |
| ≥ 4 months | 28.8 (23.8 to 33.5)** | 29.7 (21.6 to 37.0)** | 26.7 (18.5 to 34.0)** | 30.2 (8.2 to 47.0)* |
| **Booster dose (mRNA-1273)** |  |  |  |  |
| 0 – 13 days | 46.2 (42.2 to 49.9)** | 47.1 (41.9 to 51.8)** | 43.3 (36.5 to 49.4)** | 33.0 (-32.9 to 66.2)† |
| 14 – 29 days | 68.4 (66.4 to 70.3)** | 69.9 (67.2 to 72.5)** | 64.9 (61.6 to 67.9)** | 73.7 (59.6 to 82.8)** |
| 1 month | 59.3 (57.2 to 61.3)** | 58.9 (55.9 to 61.7)** | 58.8 (55.4 to 61.9)** | 57.7 (44.9 to 67.5)** |
| 2 months | 51.1 (48.2 to 53.8)** | 53.8 (50 to 57.3)** | 47.9 (42.8 to 52.6)** | 46.1 (29.6 to 58.8)** |
| 3 months | 47.1 (43.7 to 50.4)** | 49.7 (45.0 to 54.0)** | 45.5 (39.8 to 50.6)** | 42.0 (22.2 to 56.8)** |
| ≥ 4 months | 37.1 (31.5 to 42.2)** | 36.9 (26.9 to 45.5)** | 40.6 (32.6 to 47.6)** | 25.1 (-1.2 to 44.6)† |
| **2nd Booster dose (BNT162b2)** |  |  |  |  |
| 0 – 6 days | 19.7 (-35.3 to 52.3)† | 15.5 (-112.5 to 66.4)† | -27.9 (-262.9 to 54.9)† | 45.3 (-30.8 to 77.1)† |
| 7 – 29 days | 34.4 (12.5 to 50.8)* | 37.9 (0.1 to 61.4)* | 38.7 (2 to 61.6)* | 22.1 (-45.1 to 58.2)† |
| ≥ 1 month | 40.4 (28.4 to 50.4)** | 44.2 (23.5 to 59.2)** | 34.9 (12.4 to 51.7)* | 46.9 (18.7 to 65.3) |
| **2nd Booster dose (mRNA-1273)** |  |  |  |  |
| 0 – 6 days | 35.6 (-16.2 to 64.4)† | 56.5 (-19.7 to 84.2)† | 15.1 (-188.4 to 75)† | 23.2 (-100.8 to 70.6)† |
| 7 – 29 days | 40.8 (11.4 to 60.5)* | 54.0 (15.6 to 75)* | 14.6 (-95.7 to 62.7)† | 34.7 (-40.8 to 69.7)† |
| ≥ 1 month | 48.4 (32.9 to 60.3)** | 60.6 (40.6 to 73.9)** | 50.2 (23.4 to 67.6)* | 19.6 (-48.3 to 56.4) |

†p>0.05; *p≤0.05; **p<0.001 – p-values for Wald’s test two-sided.

1. Primary series with BNT162b2

| **Period** | **Overall** | **18-49 years** | **50-64 years** | **≥ 65 years** |
| --- | --- | --- | --- | --- |
| **Booster dose (BNT162b2)** |  |  |  |  |
| 0 – 13 days | 44.3 (41.9 to 46.5)** | 44.4 (42.0 to 46.8)** | 55.2 (44.2 to 64.1)** | 57.4 (19.1 to 77.6)* |
| 14 – 29 days | 62.7 (61.0 to 64.4)** | 61.9 (60.0 to 63.7)** | 67.2 (61.0 to 72.4)** | 62.2 (43.5 to 74.8)** |
| 1 month | 54.9 (53.2 to 56.6)** | 52.9 (50.9 to 54.9)** | 61.1 (55.3 to 66.1)** | 45.3 (28.3 to 58.3)** |
| 2 months | 46.2 (44.1 to 48.2)** | 44.1 (41.7 to 46.4)** | 55.3 (49.0 to 60.8)** | 33.5 (13.2 to 49.0)* |
| 3 months | 40.5 (37.7 to 43.1)** | 40.5 (37.3 to 43.5)** | 51.1 (43.8 to 57.5)** | 19.6 (-6.6 to 39.4)† |
| ≥ 4 months | 29.1 (24.8 to 33.2)** | 33.4 (28.4 to 38.2)** | 40.0 (29.9 to 48.7)** | 12.5 (-17.8 to 34.9)† |
| **Booster dose (mRNA-1273)** |  |  |  |  |
| 0 – 13 days | 48.8 (45.7 to 51.7)** | 49.7 (46.6 to 52.6)** | 46.7 (31.6 to 58.5)** | 17.2 (-92.4 to 64.4)† |
| 14 – 29 days | 66.2 (63.9 to 68.4)** | 66.3 (63.9 to 68.6)** | 62.9 (53.8 to 70.2)** | 70.1 (48.8 to 82.6)** |
| 1 month | 57.3 (55.1 to 59.5)** | 55.2 (52.5 to 57.7)** | 65.2 (58.4 to 71.0)** | 50.3 (33.9 to 62.6)** |
| 2 months | 49.6 (46.9 to 52.2)** | 50.1 (47.1 to 53.0)** | 49.2 (39.3 to 57.4)** | 42.5 (23.1 to 57)** |
| 3 months | 43.5 (39.8 to 47)** | 45.4 (41.3 to 49.2)** | 50.4 (40.1 to 58.9)** | 26.4 (-0.5 to 46)† |
| ≥ 4 months | 30.9 (24.5 to 36.7)** | 33.3 (24.1 to 41.5)** | 36.3 (21.0 to 48.5)** | 27.7 (1.2 to 47.1)* |
| **2nd Booster dose (BNT162b2)** |  |  |  |  |
| 0 – 6 days | 15.1 (-64.5 to 56.1)† | 51.6 (-103 to 88.5)† | 37.7 (-91.3 to 79.7)† | -37.5 (-312.2 to 54.1)† |
| 7 – 29 days | 50.0 (27.3 to 65.6)** | 55 (23.6 to 73.5)* | 63.6 (24.7 to 82.4)* | 33.6 (-56.1 to 71.8)† |
| ≥ 1 month | 33.0 (13.4 to 48.2)* | 25.1 (-13.2 to 50.4)† | 57.7 (35 to 72.5)** | 19.3 (-50.4 to 56.7)† |
| **2nd Booster dose**  **(mRNA-1273)** |  |  |  |  |
| 0 – 6 days | 48.6 (-16.8 to 77.4)† | 36.3 (-128.2 to 82.2)† | § | 22.1 (-183.3 to 78.6)† |
| 7 – 29 days | 61.0 (32.2 to 77.6)** | 41.1 (-55.1 to 77.6)† | 62 (-13.9 to 87.3)† | 75.9 (37.4 to 90.8)* |
| ≥ 1 month | 40.4 (11.3 to 60)* | 32.6 (-29.8 to 65)† | 64.9 (28.1 to 82.8)* | 28.4 (-56.5 to 67.3)† |

†p>0.05; *p≤0.05; **p<0.001– p-values for Wald’s test two-sided.

§ indicates a period without enough cases and controls to reliably estimate the vaccine effectiveness

# Table H. Relative Vaccine Effectiveness against severe disease in Brazil and Scotland expressed in percentages (95% CI) according to the type of vaccine used at the primary series and by age group. Reference group: individuals that received only a primary series.

1. **Brazil**
2. Primary series with ChAdOx1

| **Period** | **Overall** | **18-49 years** | **50-64 years** | **≥ 65 years** |
| --- | --- | --- | --- | --- |
| **Booster dose (BNT162b2)** |  |  |  |  |
| 0 – 13 days | 59.6 (56.3 to 62.7)** | 66.7 (60.5 to 72.0)** | 60.8 (56.2 to 64.9)** | 45.1 (35.5 to 53.3)** |
| 14 – 29 days | 79.0 (77.3 to 80.6)** | 78.3 (73.9 to 81.9)** | 79.6 (77.1 to 81.7)** | 76.0 (72.2 to 79.3)** |
| 1 month | 75.6 (74.3 to 76.9)** | 77.0 (72.9 to 80.5)** | 77.8 (75.7 to 79.7)** | 71.9 (69.7 to 73.9)** |
| 2 months | 68.2 (66.2 to 70.0)** | 75.0 (68.7 to 80.1)** | 77.5 (74.1 to 80.4)** | 64.8 (62.2 to 67.2)** |
| 3 months | 65.7 (62.5 to 68.6)** | 72.8 (62.7 to 80.2)** | 72.9 (65.5 to 78.6)** | 62.2 (58.2 to 65.8)** |
| ≥ 4 months | 59.4 (53.3 to 64.7)** | 68.8 (43.5 to 82.8)** | 55.8 (31.2 to 71.7)** | 56.2 (48.7 to 62.5)** |

†p>0.05; *p≤0.05; **p<0.001– p-values for Wald’s test two-sided.

1. Primary series with BNT162b2

| **Period** | **Overall** | **18-49 years** | **50-64 years** |
| --- | --- | --- | --- |
| **Booster dose (BNT162b2)** |  |  |  |
| 0 – 13 days | 52.6 (40.6 to 62.2)** | 45.6 (28.0 to 58.9)** | 62.4 (44.8 to 74.3)** |
| 14 – 29 days | 54.6 (43.9 to 63.2)** | 46.4 (29.4 to 59.3)** | 61.6 (46.7 to 72.4)** |
| 1 month | 58.0 (48.1 to 66.0)** | 53.2 (36.6 to 65.5)** | 61.4 (47.9 to 71.4)** |
| 2 months | 55.1 (35.3 to 68.9)** | 42.9 (1.2 to 67.0)* | 56.7 (28.5 to 73.8)* |
| 3 months | 60.8 (18.0 to 81.3)* | 85.4 (−8.1 to 98.0)† | 34.9 (−51.5 to 72.0)† |
| ≥ 4 months | 38.2 (−125.0 to 83.0)† | § | § |

†p>0.05; *p≤0.05; **p<0.001– p-values for Wald’s test two-sided.

§ indicates a period without enough cases and controls to reliably estimate the vaccine effectiveness

1. **Scotland**

|  | **ChAdOx1** | **BNT162b2** |
| --- | --- | --- |
| **Period** | **Overall** | **Overall** |
| **Booster dose (BNT162b2)** |  |  |
| 0 – 13 days | 72.2 (34.5 to 88.2)* | 40.9 (-53.5 to 77.3)† |
| 14 – 29 days | 85.2 (67.3 to 93.3)** | 78.2 (27.7 to 93.4)* |
| 1 month | 78.5 (65.2 to 86.7)** | 65.3 (33.2 to 81.9)* |
| 2 months | 63.6 (44.1 to 76.3)** | 63.1 (33.8 to 79.4)** |
| 3 months | 58.9 (32.3 to 75.0)** | 60.9 (23.1 to 80.1)* |
| ≥ 4 months | 54.9 (14.2 to 76.3)* | 39.7 (-28.6 to 71.7)† |
| **Booster dose (mRNA-1273)** |  |  |
| 0 – 13 days | 89.8 (25.4 to 98.6)* | § |
| 14 – 29 days | 95.5 (67.3 to 99.4)* | 78.3 (-60.8 to 97.1)† |
| 1 month | 83.6 (66.0 to 92.1)** | 72.9 (20.3 to 90.8)* |
| 2 months | 80.1 (59.3 to 90.2)** | 78.3 (25.9 to 93.6)* |
| 3 months | 67.5 (29.0 to 85.1)* | 75.6 (-8.0 to 94.5)† |
| ≥ 4 months | 59.3 (3.6 to 82.8)* | 66.2 (-60.6 to 92.9)† |

†p>0.05; *p≤0.05; **p<0.001– p-values for Wald’s test two-sided.

§ indicates a period without enough cases and controls to reliably estimate the vaccine effectiveness

# Table I. Vaccine Effectiveness against symptomatic infection among individuals with a previously confirmed infection in Brazil compared to unvaccinated. Results were reported as percentages (95% CI), according to the type of vaccine used in the primary series and by age group.

1. Primary series with ChAdOx1

| **Period** | **Overall** | **18-49 years** | **50-64 years** | **≥ 65 years** |
| --- | --- | --- | --- | --- |
| **Booster dose (BNT162b2)** |  |  |  |  |
| 0 – 13 days | 57.4 (55.4 to 59.4)** | 57.3 (54.7 to 59.7)** | 60.4 (56.6 to 63.9)** | 62.7 (51.8 to 71.2)** |
| 14 – 29 days | 71.9 (70.5 to 73.2)** | 70.1 (68.3 to 71.8)** | 75.7 (73.4 to 77.8)** | 81.0 (76.0 to 85.0)** |
| 1 month | 61.6 (59.9 to 63.2)** | 58.6 (56.3 to 60.8)** | 67.7 (64.8 to 70.3)** | 70.7 (65.4 to 75.2)** |
| 2 months | 48.0 (45.1 to 50.7)** | 44.1 (40.1 to 47.8)** | 58.2 (53.7 to 62.3)** | 55.3 (46.7 to 62.4)** |
| 3 months | 41.9 (37.5 to 45.9)** | 36.6 (30.5 to 42.1)** | 54.3 (47.0 to 60.6)** | 50.7 (37.7 to 61.0)** |
| ≥ 4 months | 24.9 (14.8 to 33.7)** | 26.8 (13.9 to 37.8)** | 22.0 (0.0 to 39.2)* | 33.2 (4.5 to 53.2)* |

†p>0.05; *p≤0.05; **p<0.001– p-values for Wald’s test two-sided.

1. Primary series with BNT162b2

| **Period** | **Overall** | **18-49 years** | **50-64 years** |
| --- | --- | --- | --- |
| **Booster dose (BNT162b2)** |  |  |  |
| 0 – 13 days | 58.5 (55.4 to 61.4)** | 57.5 (53.9 to 60.9)** | 62.7 (56.2 to 68.3)** |
| 14 – 29 days | 62.2 (59.1 to 65.0)** | 59.6 (55.8 to 63.1)** | 70.1 (64.6 to 74.7)** |
| 1 month | 55.2 (51.2 to 58.9)** | 52.4 (47.4 to 56.9)** | 63.5 (56.5 to 69.4)** |
| 2 months | 44.3 (35.1 to 52.2)** | 35.2 (22.7 to 45.7)** | 65.2 (52.4 to 74.6)** |
| 3 months | 24.7 (−2.7 to 44.8)† | 22.5 (−12.9 to 46.9)† | 35.4 (−15.3 to 63.8)† |
| ≥ 4 months | 49.8 (−27.1 to 80.1)† | § | § |

†p>0.05; *p≤0.05; **p<0.001– p-values for Wald’s test two-sided.

§ indicates a period without enough cases and controls to reliably estimate the vaccine effectiveness

# Fig A. Distribution of variants of concern in Brazil and Scotland over time

## Brazil


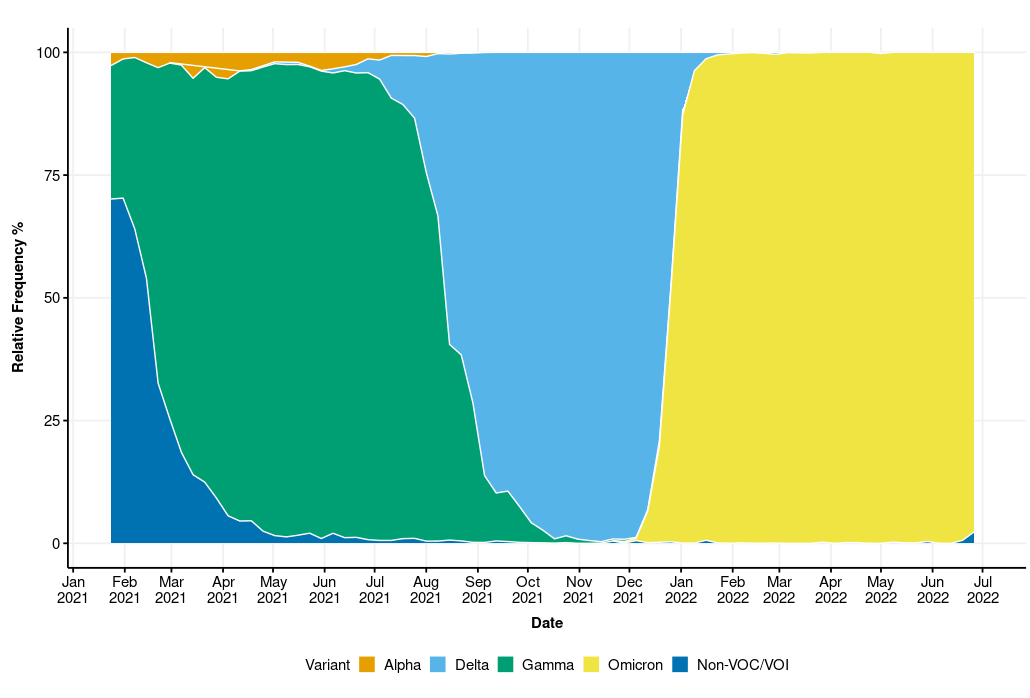


## Scotland


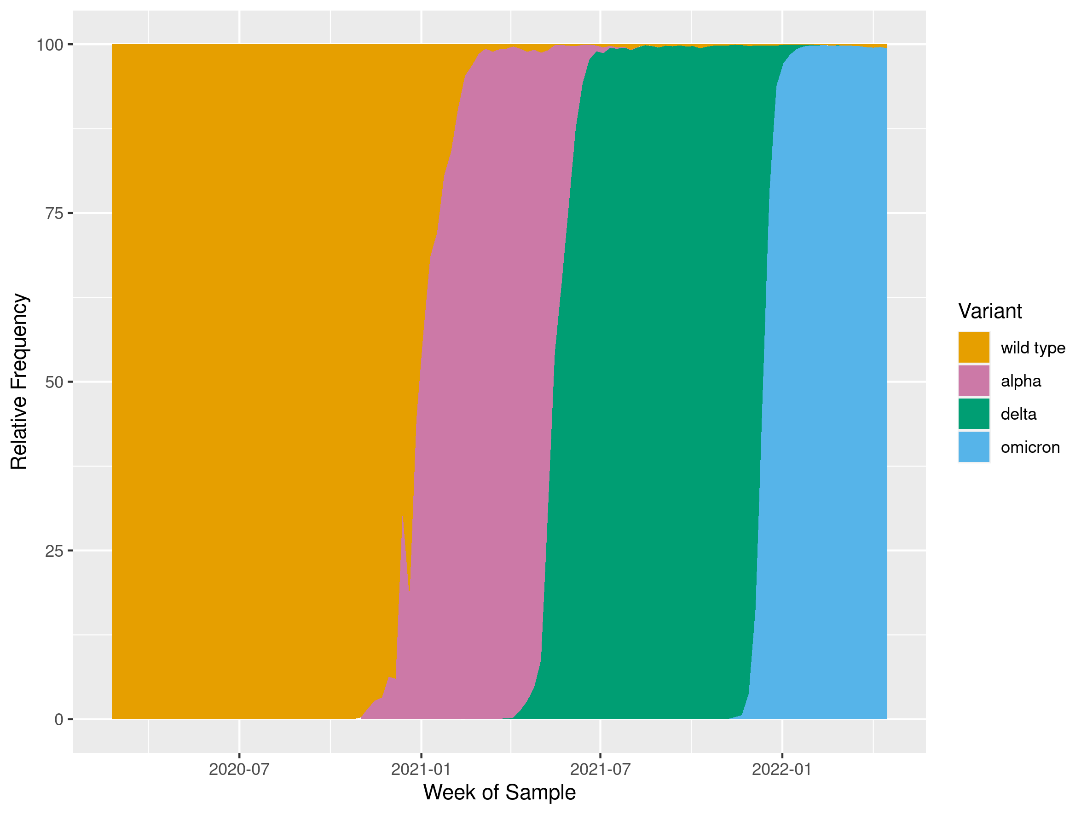


# Fig B. STROBE Flowchart of the study population in Brazil (A) and Scotland (B)

##
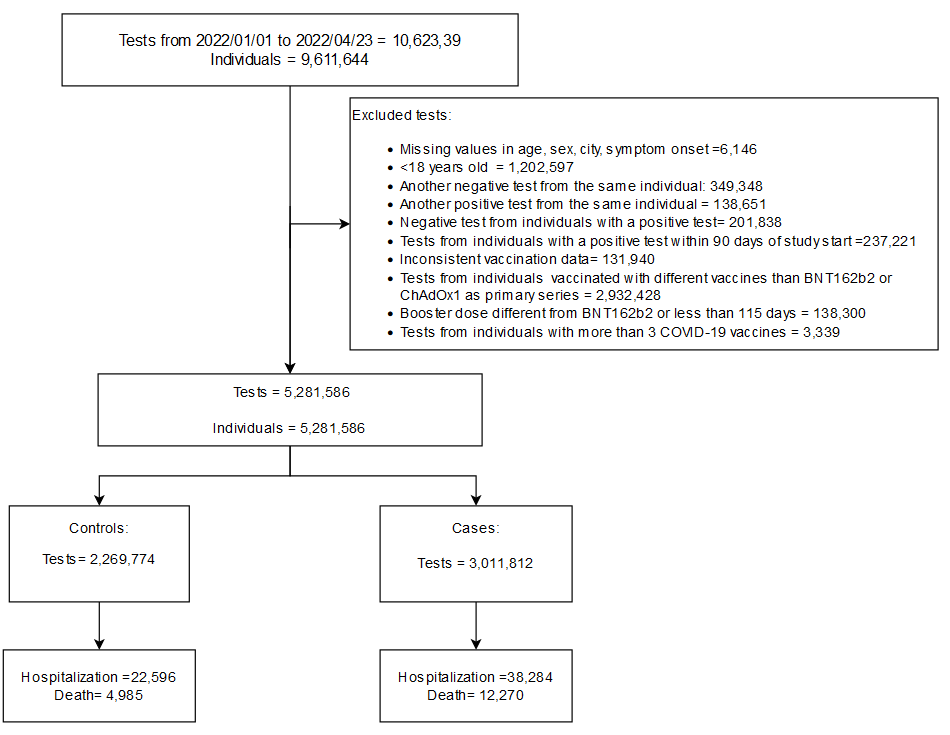
Brazil

## Scotland


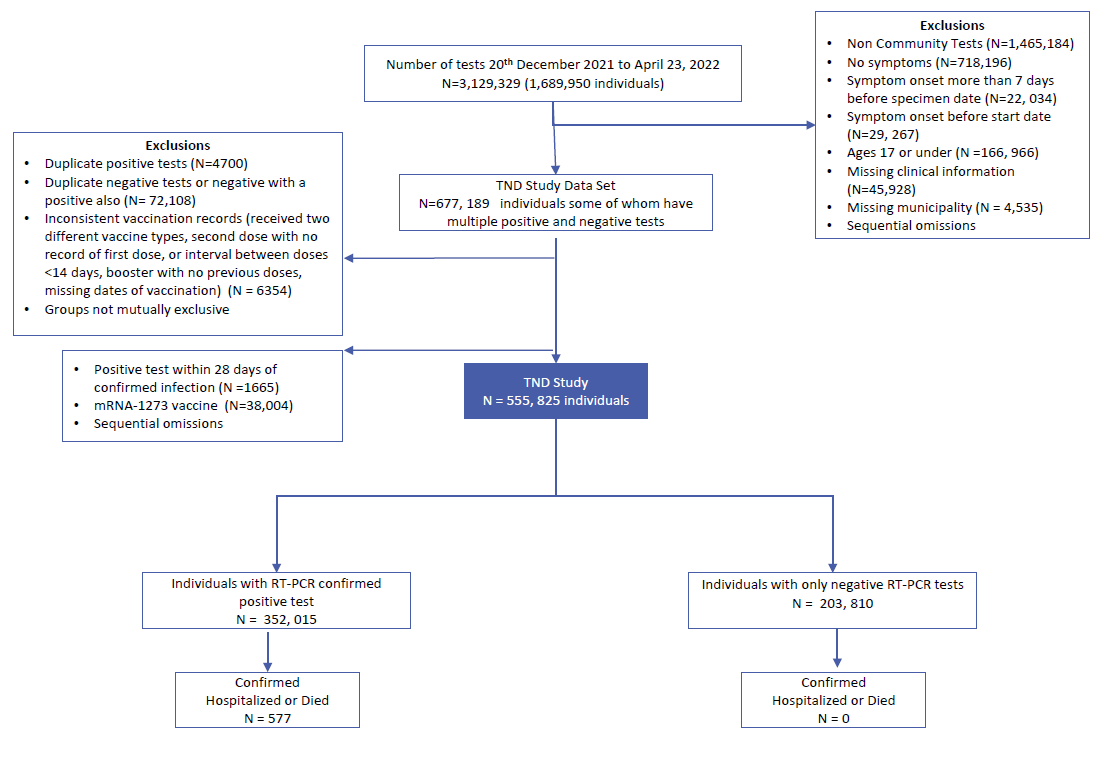


TND = Test Negative Design; RT-PCR = Real-Time Polymerase Chain Reaction

# Fig C. Distribution case and control over time in each country for individuals unvaccinated or vaccinated with ChAdOx1 or BNT162b2 as primary series.

## Brazil


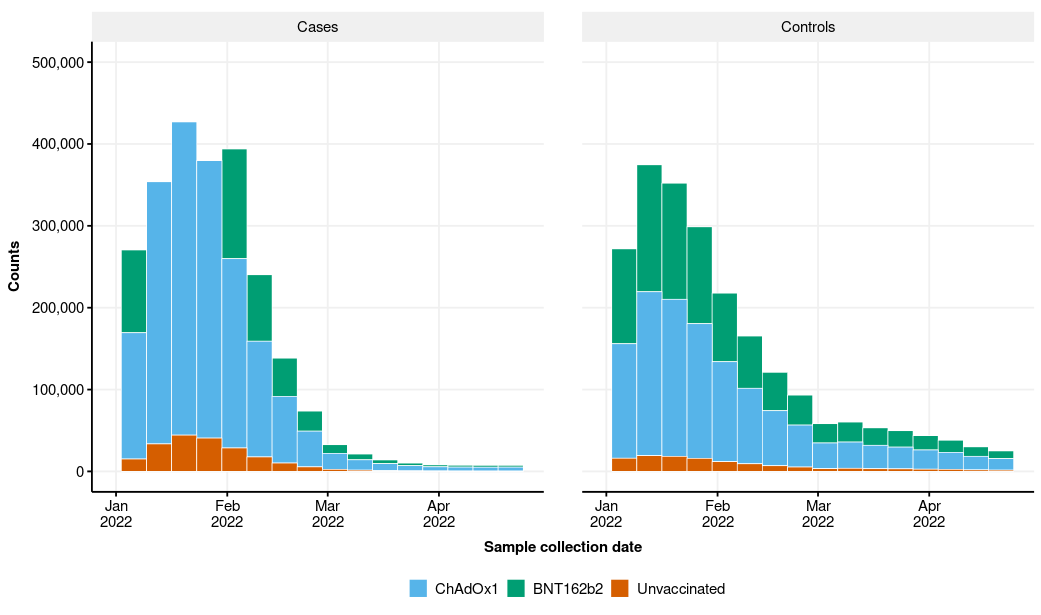


## Scotland


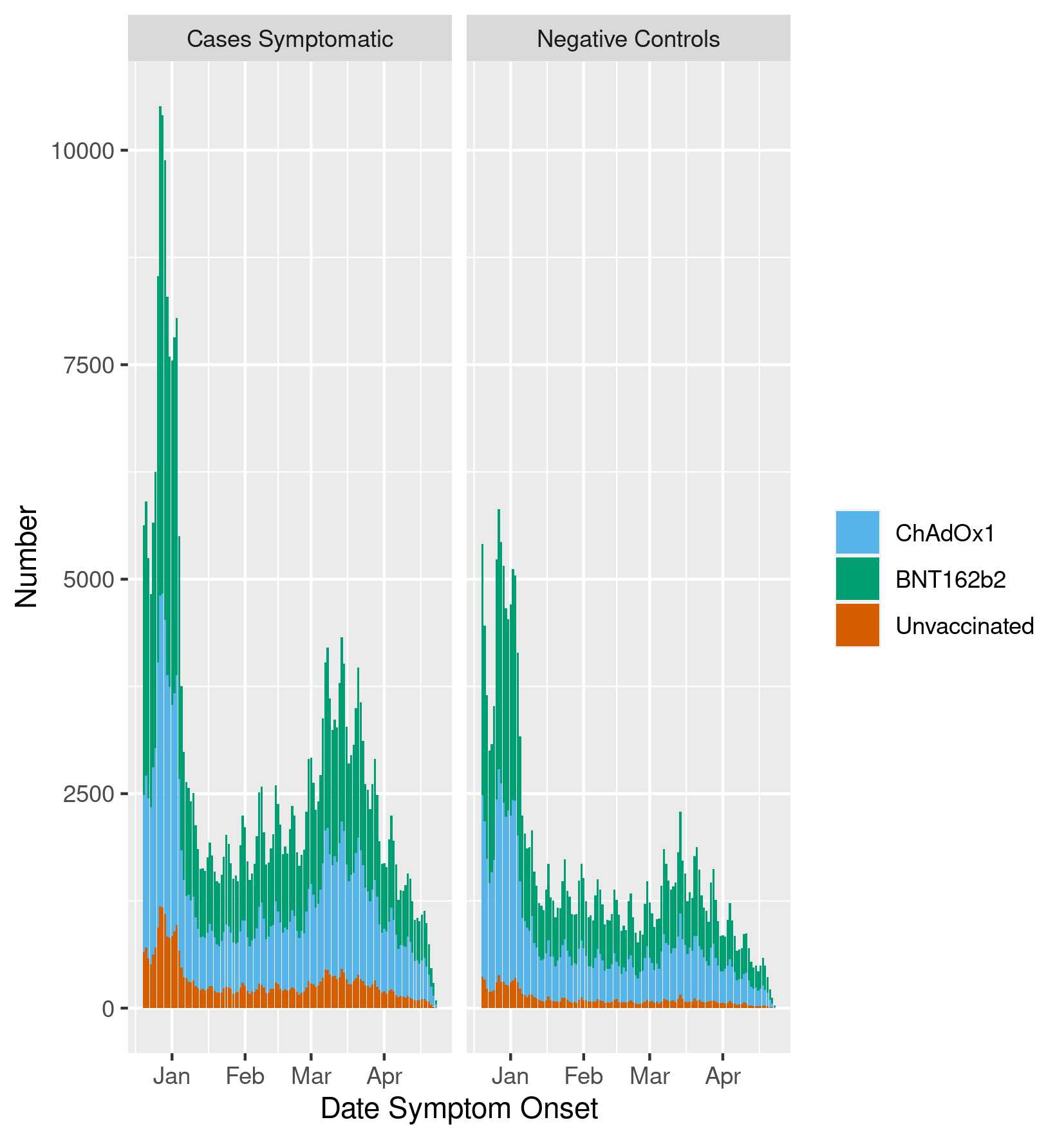


# Fig D. Uptake of each dose, including booster dose, in individuals vaccinated with ChAdOx1 or BNT162b2 as primary series. Vaccination numbers in Brazil (A) and Scotland (B), stratified by age group and primary series. Different Y-axis scales in each age group.

## Brazil


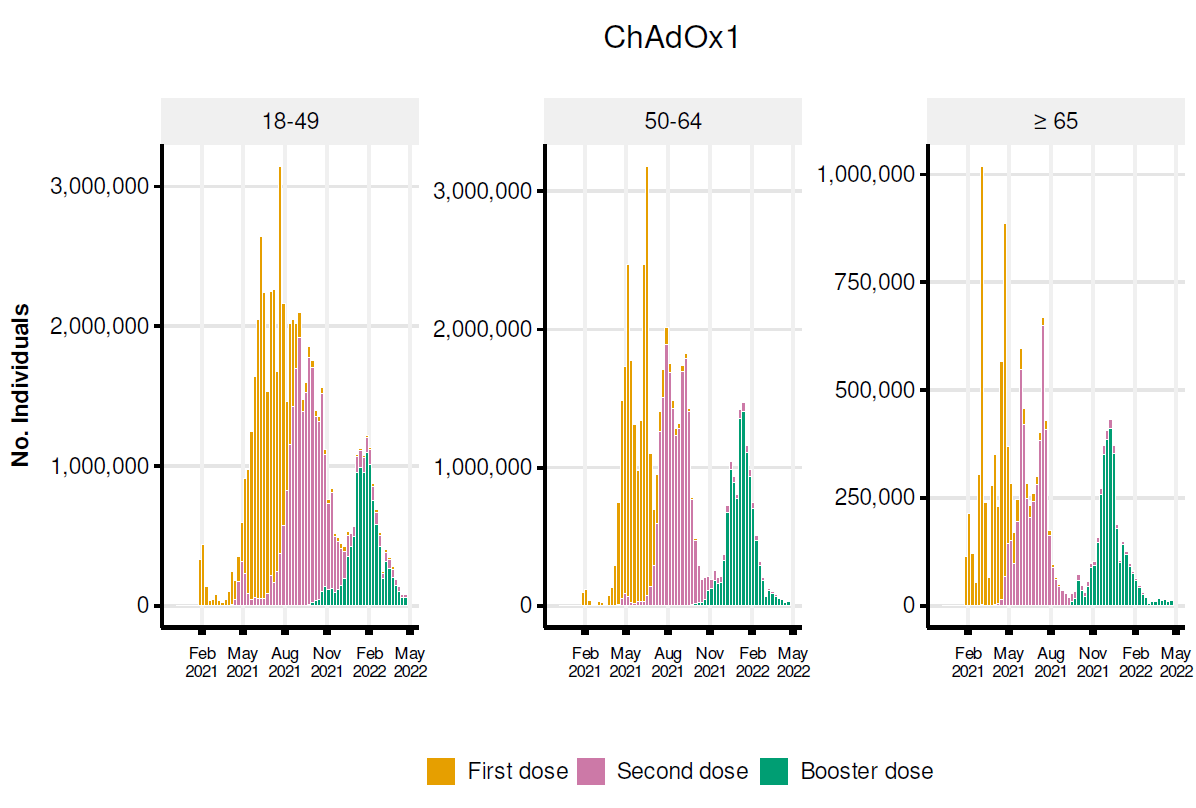


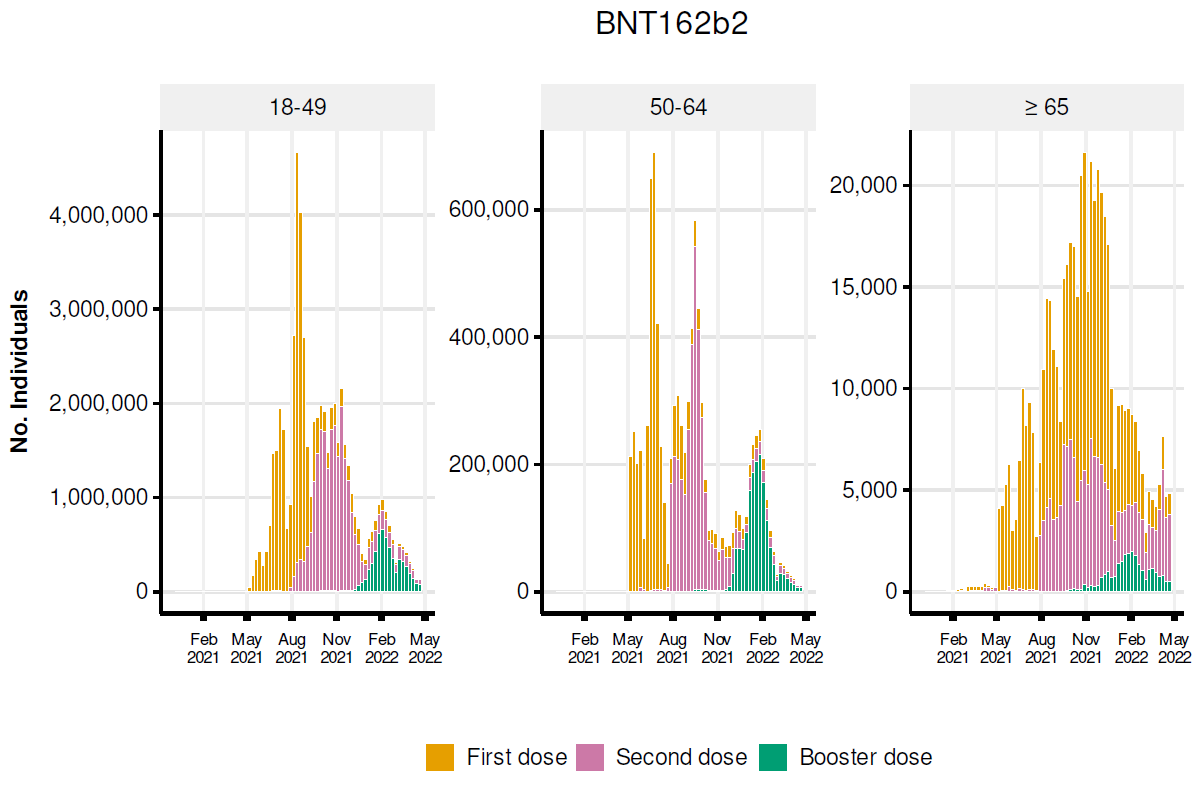


## Scotland


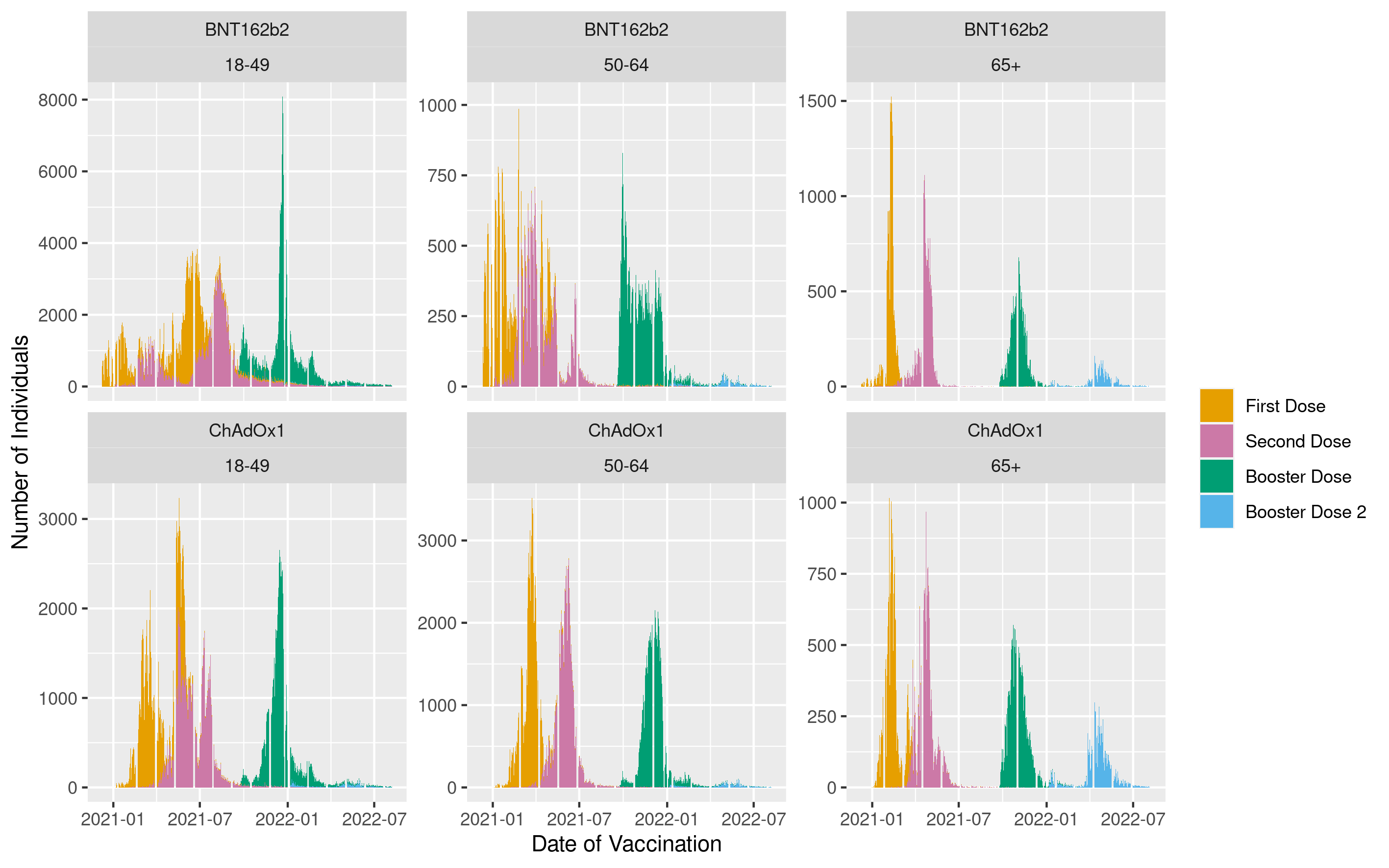


# References

1. Clift AK, Coupland CAC, Keogh RH, Diaz-Ordaz K, Williamson E, Harrison EM, et al. Living risk prediction algorithm (QCOVID) for risk of hospital admission and mortality from coronavirus 19 in adults: national derivation and validation cohort study. BMJ. 2020;371: m3731. doi:10.1136/bmj.m3731
