# Supplementary material for: Effectiveness of mRNA boosters after homologous primary series with BNT162b2 or ChAdOx1 against symptomatic infection and severe COVID-19 in Brazil and Scotland: A test-negative design case–control study
Source: PLoS Med. 2023 Jan 11;20(1):e1004156. doi: 10.1371/journal.pmed.1004156 (PMC9879484; doi:10.1371/journal.pmed.1004156)
Supplement: S2 Appendix — (DOCX) [file pmed.1004156.s002.docx]

# S3-APPENDIX. STROBE/RECORD checklist

|  | **Item No.** | **STROBE items** | **RECORD items** | **Location in manuscript where items are reported** |
| --- | --- | --- | --- | --- |
| **Title and abstract** | | |  |  |
|  | 1 | (a) Indicate the study’s design with a commonly used term in the title or the abstract (b) Provide in the abstract an informative and balanced summary of what was done and what was found | RECORD 1.1: The type of data used should be specified in the title or abstract. When possible, the name of the databases used should be included.  RECORD 1.2: If applicable, the geographic region and timeframe within which the study took place should be reported in the title or abstract.  RECORD 1.3: If linkage between databases was conducted for the study, this should be clearly stated in the title or abstract. | 1.1. The type of data is described, and details in the Methods in the section “Study design, population and data sources”  1.2. Time and region described in the Abstract and methods (“Study design, population and data sources”)  1.3. Linkage of databases pointed in the methods (“Study design, population and data sources”) |
| **Introduction** | | |  |  |
| Background rationale | 2 | Explain the scientific  background and rationale for the investigation being reported |  | Introduction Section, paragraphs 2 and 3 |
| Objectives | 3 | State specific objectives, including any prespecified hypotheses |  | Introduction Section, paragraph 3 |
| **Methods** | | |  |  |
| Study Design | 4 | Present key elements of study design early in the paper |  | Methods paragraph 1 |
| Setting | 5 | Describe the setting, locations, and relevant dates, including periods of recruitment, exposure, follow-up, and data collection |  | Methods paragraphs 1-6 |
| Participants | 6 | 1. Cohort study - Give the eligibility criteria, and the sources and methods of selection of participants. Describe methods of follow-up   Case-control study - Give the eligibility criteria, and the sources and methods of case ascertainment and control selection. Give the rationale for the choice of cases and controls Cross-sectional study - Give the eligibility criteria, and the sources and methods of selection of participants     1. Cohort study - For matched studies, give matching criteria and number of exposed and unexposed   Case-control study - For matched studies, give matching criteria and the number of controls per case | RECORD 6.1: The methods of study population selection (such as codes or algorithms used to identify subjects) should be listed in detail. If this is not possible, an explanation should be provided.    RECORD 6.2: Any validation studies of the codes or algorithms used to select the population should be referenced. If validation was conducted for this study and not published elsewhere, detailed methods and results should be provided.    RECORD 6.3: If the study involved linkage of databases, consider use of a flow diagram or other graphical display to demonstrate the data linkage process, including the number of individuals with linked data at each stage. | 6.1 – Informations were included in:  Methods paragarph 1-6  6.2.NA  6.3. Previously described in “Katikireddi, Srinivasa Vittal, et al. "Two-dose ChAdOx1 nCoV-19 vaccine protection against COVID-19 hospital admissions and deaths over time: a retrospective, population-based cohort study in Scotland and Brazil." *The Lancet* 399.10319 (2022): 25-35.” [1] |
| Variables | 7 | Clearly define all outcomes, exposures, predictors, potential confounders, and effect modifiers. Give diagnostic criteria, if applicable. | RECORD 7.1: A complete list of codes and algorithms used to classify exposures, outcomes, confounders, and effect modifiers should be provided. If these cannot be reported, an explanation should be provided. | Methods paragraphs 5-7 |
| Data sources/ measurement | 8 | For each variable of interest, give sources of data and details of methods of assessment (measurement).  Describe comparability of assessment methods if there is  more than one group |  | Methods paragraphs 8,9 |
| Bias | 9 | Describe any efforts to address potential sources of bias |  | Methods paragraph 9 |
| Study size | 10 | Explain how the study size was arrived at |  | Methods paragraph 4,5 |
| Quantitative variables | 11 | Explain how quantitative variables were handled in the analyses. If applicable, describe which groupings were chosen,  and why |  | Methods paragraph 8,9 |
| Statistical methods | 12 | (a) Describe all statistical methods, including those used to control for confounding (b) Describe any methods used to examine subgroups and interactions   1. Explain how missing data were addressed 2. Cohort study - If applicable, explain how loss to follow-up was addressed   Case-control study - If applicable, explain how matching of cases and controls was addressed  Cross-sectional study - If applicable, describe analytical methods taking account of sampling strategy   1. Describe any sensitivity analyses |  | (a) Methods paragraph 8,9  (b) Methods paragraph 1-7  (c) Methods paragraph 8,9  (d) Not applicable to the study design  (e) Methods paragraph 9 |
| Data access and cleaning methods |  |  | RECORD 12.1: Authors should describe the extent to which the investigators had access to the database population used to create the study population. | Methods paragraph 4,5 |
|  |  |  | RECORD 12.2: Authors should provide information on the data cleaning methods used in the study. | Previously described in “Katikireddi, Srinivasa Vittal, et al. "Two-dose ChAdOx1 nCoV-19 vaccine protection against COVID-19 hospital admissions and deaths over time: a retrospective, population-based cohort study in Scotland and Brazil." *The Lancet* 399.10319 (2022): 25-35.” [1] |
| Linkage |  |  | RECORD 12.3: State whether the study included person-level,  institutional-level, or other data linkage across two or more databases. The methods of linkage and methods of linkage quality evaluation should be provided. | Previously described in “Katikireddi, Srinivasa Vittal, et al. "Two-dose ChAdOx1 nCoV-19 vaccine protection against COVID-19 hospital admissions and deaths over time: a retrospective, population-based cohort study in Scotland and Brazil." *The Lancet* 399.10319 (2022): 25-35.” [1] |
| Participants | 13 | 1. Report the numbers of individuals at each stage of the study (e.g., numbers potentially eligible, examined for eligibility, confirmed eligible, included in the study, completing follow-up, and analysed) 2. Give reasons for nonparticipation at each stage. (c) Consider use of a flow diagram | RECORD 13.1: Describe in detail the selection of the persons included in the study (i.e., study population selection) including filtering based on data quality, data availability and linkage. The selection of included persons can be described in the text and/or by means of the study flow diagram. | S1. Appendix S2 Figure |
| Descriptive data | 14 | 1. Give characteristics of study participants (e.g., demographic, clinical, social) and information on exposures and potential confounders 2. Indicate the number of participants with missing data for each variable of interest (c) Cohort study - summarise follow-up time (e.g., average and total amount) |  | In the Results paragraph 1 and supplementary table S1 |
| Outcome data | 15 | Cohort study - Report numbers of outcome events or summary measures over time  Case-control study - Report numbers in each exposure category, or summary measures of exposure  Cross-sectional study - Report numbers of outcome events or summary measures |  | Provided as a Table 1 and S1. Appendix S2 Figure |
| Main results | 16 | (a) Give unadjusted estimates and, if applicable, confounderadjusted estimates and their precision (e.g., 95% confidence interval). Make clear which confounders were adjusted for and why they were included (b) Report category boundaries when continuous variables were categorized  (c) If relevant, consider translating estimates of relative risk into absolute risk for a meaningful time period |  | In the Results paragraphs 2-10 |
| Other analyses | 17 | Report other analyses done—  e.g., analyses of subgroups and interactions, and sensitivity analyses |  | Sensitivity and Subgroups analyses are presented in the results section paragraphs 5,6,8-10 |
| Key results | 18 | Summarise key results with reference to study objectives |  | Summarised both in abstracts and results |
| Limitations | 19 | Discuss limitations of the study, taking into account sources of potential bias or imprecision. Discuss both direction and magnitude of any potential bias | RECORD 19.1: Discuss the  implications of using data that were not created or collected to answer the specific research question(s). Include discussion of misclassification bias, unmeasured confounding, missing data, and changing eligibility over time, as they pertain to the study being reported. | In discussion section paragraph 8 |
| Interpretation | 20 | Give a cautious overall interpretation of results considering objectives, |  | Discussion paragraph 1,9 |
|  |  | limitations, multiplicity of analyses, results from similar studies, and other relevant evidence |  | Discussion paragraph 8 |
| Generalisability | 21 | Discuss the generalisability (external validity) of the study results |  | Discussion paragraph 7 |
| Funding | 22 | Give the source of funding and the role of the funders for the present study and, if applicable, for the original study on which the present article is based |  | Acknowledgements section paragraph 1 |
| Accessibility of protocol, raw data, and programming code |  |  | RECORD 22.1: Authors should provide information on how to access any supplemental information such as the study protocol, raw data, or programming code. | These information are available at: <https://vigivac.fiocruz.br> – except for raw data, that is protected under Brazilian personal data protection law (LGPD), as explained in Data availability section |

# References

1. Katikireddi SV, Cerqueira-Silva T, Vasileiou E, Robertson C, Amele S, Pan J, et al. Two-dose ChAdOx1 nCoV-19 vaccine protection against COVID-19 hospital admissions and deaths over time: a retrospective, population-based cohort study in Scotland and Brazil. The Lancet. 2022;399: 25–35. doi:10.1016/S0140-6736(21)02754-9
