# Supplementary material for: Effectiveness of mRNA boosters after homologous primary series with BNT162b2 or ChAdOx1 against symptomatic infection and severe COVID-19 in Brazil and Scotland: A test-negative design case–control study
Source: PLoS Med. 2023 Jan 11;20(1):e1004156. doi: 10.1371/journal.pmed.1004156 (PMC9879484; doi:10.1371/journal.pmed.1004156)
Supplement: S3 Appendix — (DOCX) [file pmed.1004156.s003.docx]

Analysis plan to investigate potential waning of COVID-19 vaccine protection after a booster dose.

| **Full Project Title** |  |
| --- | --- |
| **Version Number** | V1 |
| **Previous Versions** |  |
| **Effective Date** |  |
| **Analyst(s)** | Chris Robertson (CR), Thiago Cerqueira-Silva (TCS), |
| **Co-authors** |  |
| **Approved** |  |

| **Version History** | | | |
| --- | --- | --- | --- |
| **Version** | **Date** | **Author** | **Notes** |
| V1 | 03/02/2022 | TCS |  |
| V2 | 14/02/2022 | TCS | Update in the categories for vaccination status |
| V3 |  |  |  |
|  |  |  |  |
|  |  |  |  |

Contents

[Introduction](#_30j0zll) **2**

[Aims and Objectives](#_1fob9te) **3**

[2.1 Aims](#_3znysh7) [3](#_3znysh7)

[2.2 Objectives](#_2et92p0) [3](#_2et92p0)

[Study Design](#_4d34og8) **3**

[3.1 Study design](#_2s8eyo1) [3](#_2s8eyo1)

[3.2 Setting](#_17dp8vu) [3](#_17dp8vu)

[3.3 Population](#_3rdcrjn) [3](#_3rdcrjn)

[3.4 Data sources](#_26in1rg) [3](#_26in1rg)

[3.5 Inclusion/exclusion criteria](#_lnxbz9) [3](#_lnxbz9)

[3.6 Sample size calculations](#_35nkun2) [3](#_35nkun2)

[Data and Data Validation](#_1ksv4uv) **5**

[4.1 Data variables available](#_44sinio) [5](#_44sinio)

[4.2 Constructed variables](#_2jxsxqh) [5](#_2jxsxqh)

[4.3 Consistency and error checking](#_z337ya) [5](#_z337ya)

[Statistical analyses](#_3j2qqm3) **6**

[5.1 Objective a) Assess the effectiveness of the vaccines](#_vx1227) [6](#_vx1227)

[5.1.1 Exposures of interest](#_1y810tw) [6](#_1y810tw)

[5.1.2 Outcomes of interest](#_2xcytpi) [6](#_2xcytpi)

[5.1.3 Potential confounders](#_1ci93xb) [6](#_1ci93xb)

[5.1.4 Potential effect modifiers](#_3whwml4) [6](#_3whwml4)

[5.1.5 Analytical techniques](#_qsh70q) [6](#_qsh70q)

[5.1.6 Sub-group analysis](#_3as4poj) [7](#_3as4poj)

[5.1.7 Corrections for multiple testing](#_1pxezwc) [7](#_1pxezwc)

[5.1.8 Sensitivity analysis](#_49x2ik5) [7](#_49x2ik5)

[5.1.9 Other analysis](#_2p2csry) [7](#_2p2csry)

[5.2 Missing data](#_147n2zr) [7](#_147n2zr)

[5.3 Statistical software](#_3o7alnk) [7](#_3o7alnk)

[Reporting Results](#_23ckvvd) **7**

[6.1 Reporting guidelines and conventions](#_ihv636) [7](#_ihv636)

[6.2 Dissemination](#_32hioqz) [7](#_32hioqz)

[References](#_1hmsyys) **8**

[Appendix](#_41mghml) **8**

# Introduction

This study aims to determine whether protection from COVID-19 vaccination wanes after the booster dose of SARS-CoV-2 vaccines. If possible, we plan to conduct harmonised analyses across different contexts to strengthen confidence that trends in variants (VOCs) do not drive findings, increase statistical power, and allow robustness of results when adjusting for differing confounders to be assessed. A current collaboration will enable analyses to be conducted in Scotland and Brazil.

In Scotland, the primary series (two doses) mainly included Oxford-AstraZeneca (ChAdOx1) or Pfizer-BioNTech (BNT162b2). The primary series in Brazil included: ChAdOx1, CoronaVac, BNT162b2 and Ad26.COV2.S. At present, the vaccination programme in Scotland uses two mRNA vaccines as boosters: Pfizer-BioNTech (BNT162b2) and Moderna (mRNA-1273) vaccines. The vaccination programme in Brazil currently offers mainly BNT162b2 as a booster dose of a primary series with BNT162b2, ChAdOx1, Ad26.COV2.S or CoronaVac. In this study we will analyse data of BNT162b2 and ChAdOx1 as a primary series and booster with BNT162b2.

We aim to evaluate the real-world impacts of being fully vaccinated and assess whether the rate of infection and severe outcomes (deaths and hospitalisations) (RR) diminishes over time after the booster dose. e will use deidentified individual-level linked data. All data and analyses will be hosted within secure analytical environments.

# Aims and Objectives

## 2.1 Aims

To assess the relationship between time since the maximum protection following a booster dose and the risk of severe COVID-19 (hospitalisation or death). We will also assess the secondary outcomes of confirmed SARS-CoV-2 infection.

## 2.2 Objectives

By vaccine type, we seek to:

1. Estimate Odds Ratio (OR) against the composite outcome of COVID-19 hospitalisation/death of individuals with booster dose versus individuals without booster dose.
2. Estimate Odds Ratio (OR) against confirmed SARS-CoV-2 infection of individuals with booster dose versus individuals without booster dose.
3. Estimate Rate Ratio (RR) against the composite outcome of COVID-19 hospitalisation/death as a function of time post maximum effect of the booster dose of a COVID-19 vaccine.
4. Estimate RR against the separate outcomes of i) COVID-19 hospitalisation; ii) COVID-19 death as a function of time post maximum effect of the booster dose of a COVID-19 vaccine.
5. Assess if waning of protection from booster dose differs by age group, sex and being in a high-risk COVID-19 group

# Study Design

## 3.1 Study design

Objectives a) and b) will be evaluated through a test-negative design case-control study

Objective c) and d) open prospective cohort study comparing outcomes amongst vaccinated people for differing lengths of time.

## 3.2 Settings

Scotland and Brazil.

## 3.3 Population

In Scotland our main population of interest is adults who have completed a full COVID-19 vaccination schedule (typically two doses) resident in the countries. Our secondary population of interest in Scotland is all adults.

In Brazil our primary population of interest is all adults who have received a booster dose, with primary series of any available vaccine.

For the test-negative design, in both countries, we will study all adults who have received a COVID-19 RT-PCR or Rapid Antigen^^[[1]](#footnote-0)^^ test after the begin of booster dose campaign in each country (Brazil: 2021/09/15; Scotland: 2021/09/15)

## 3.4 Data sources

In Scotland, we will draw on the following databases:

- Primary care data: General Practices (n=940) for information on demographics, other population characteristics and vaccination data.
- Vaccination data: Vaccines administered in national vaccination centres and data available via the Turas Vaccination Management Tool (TVMT).
- Secondary care data: Hospital admissions through the Scottish Morbidity Record (SMR) and Rapid Preliminary Inpatient Data (RAPID).
- Laboratory test data: RT-PCR laboratory confirmed SARS-CoV-2 infection and data available via the Electronic Communication of Surveillance in Scotland (ECOSS) database.
- Mortality data from National Records Scotland (NRS)

Equivalent datasets will be used for other UK/international countries. In Brazil, we will analyse deterministically linked data provided by the Ministry of Health:

- COVID-19 vaccination (SI-PNI): Dates and types of vaccination administered, primary reason for vaccination, indigenous status, pregnancy status.
- Acute Respiratory Infection Suspected Cases (eSUS-Notifica): register of suspected and confirmed COVID-19 cases
- Severe Acute Respiratory Infection/Illness (SIVEP-Gripe): contains all hospitalisations and deaths due to severe acute respiratory illnesses, including COVID-19.

The above three databases all have whole population coverage, with returns from both public and private healthcare systems. However, data quality may vary geographically (typically data quality is better in the south of Brazil). Testing capacity has also improved over the course of the pandemic, but most improvements occurred prior to the vaccination programmes starting. In addition to the above datasets, it may be possible in the future to obtain linkage to general hospital records for patients treated within the public healthcare system (covering approximately 70% of hospitalisations in Brazil).

## 3.5 Inclusion/exclusion criteria

Inclusion criteria for the primary cohort:

- Receive at least two doses of BNT162b2 or ChAdOx1 before or during the study period.

Exclusion criteria for the primary cohort:

- Inconsistent vaccination records (i.e. received a second dose with no record of a first dose, received doses from different vaccine types or interval between doses of less than 14 days)
- Missing essential covariates (sex, age, geography)
- Children (under the age of 18 years)
- Hospitalization previous to the study start date
- A positive test within 28 days of the study start date

Inclusion criteria for the TND:

- Receive at least two doses of BNT162b2 or ChAdOx1 at the sample collection date.

Exclusion criteria for the TND:

- Tests within 90 days of a previous positive test
- Negative tests followed by a positive test within 7 days
- Negative tests within 14 days of a previous negative test
- Missing essential covariates (sex, age, geography)
- Children (under the age of 18 years)

## 3.6 Sample size calculations

Based on a preliminary review of descriptive statistics in both countries, we anticipate the following numbers:

- Scotland:
  - ChAdOx1:
  - BNT162b2:
- Brazil
  - ChAdOx1: 10 million individuals with booster from 49M with 2 doses
  - BNT162b2: 5 million individuals with booster from 28M with 2 doses

If adequate numbers are not available for statistical analysis for a specific vaccine, we will not progress that analysis and censor individuals who receive that vaccine.

# Data and Data Validation

## 4.1 Data variables available

Tables 1 and 2 lists the groupings of variables available for this study by data source. Exposure data are described in the vaccinations category. Outcome data are described in the secondary care, mortality and laboratory tests categories. The rest of categories contain data on potential confounding factors and effect modifiers.

**Table 1: Data items/variables and data sources**

| **Data category** | **Data item** | **Data source** |
| --- | --- | --- |
| Demographic | Sex | GP |
|  | Age | GP |
| Socioeconomic | SIMD | GP |
| Other characteristics | Body Mass Index (BMI) | GP |
|  | Smoking | GP |
|  | Blood Pressure | GP |
| Geographic | Urban Rural Index (UR6); Health Board | GP |
| Clinical diagnoses | Underlying conditions  (e.g., asthma, cardiac disease, immunosuppression etc.) | GP |
| Vaccinations | Vaccine type | GP, TVMT |
|  | Vaccine dose | GP, TVMT |
|  | Vaccination date | GP, TVMT |
| Laboratory tests | RT-PCR positive SARS-CoV-2 | ECOSS |
|  | RT-PCR negative SARS-CoV-2 | ECOSS |
|  | Date of RT-PCR test | ECOSS |
| Genomic Vigilance | Date of prevalence of variants | GISAID |
| Secondary care | Hospital admissions | RAPID/SMR |
| Mortality | Death with COVID-19 as the main cause according to death certificate, or death within 28 days of a positive RT-PCR test for COVID-19 | NRS |
| Abbreviations: Scottish Index of Multiple Deprivation (SIMD), Body Mass Index (BMI), Electronic Communication of Surveillance in Scotland (ECOSS), Reverse-transcription polymerase chain reaction (RT-PCR); Turas Vaccination Management Tool (TVMT); Scottish Morbidity Record (SMR); National Records of Scotland (NRS) | | |

**Table 2: Data items/variables and data sources in Brazil**

| **Data category** | **Data item** | **Data source** |
| --- | --- | --- |
| Sociodemographic | Sex | SI-PNI |
|  | Age | SI-PNI |
|  | Municipality-level IBP | SI-PNI |
|  | Education | (SIVEP-Gripe) |
|  | Race | SI-PNI, eSUS, SIVEP-Gripe |
|  | City |  |
| Clinical diagnoses | Underlying conditions  (Cardiac disease, chronic kidney disease, etc) | eSUS, SIVEP-Gripe |
| Vaccinations | Vaccine type | SI-PNI |
|  | Vaccine dose | SI-PNI |
|  | Vaccination date | SI-PNI |
|  | The primary reason for vaccination: 2 categories (comorbidities/other) | SI-PNI |
| Genomic Vigilance | Date of prevalence of variants | GISAID |
| Laboratory tests | Date of RT-PCR/rapid antigen test | eSUS |
|  | RT-PCR/rapid antigen positive SARS-CoV-2 | eSUS |
|  | RT-PCR/rapid antigen negative SARS-CoV-2 | eSUS |
| Secondary care  Mortality | COVID-19 hospital admission date | SIVEP-Gripe |
|  | COVID-19 death | SIVEP-Gripe/eSUS |
|  | COVID-19 death date | SIVEP-Gripe/eSUS |

## 4.2 Constructed variables

1. Previous infection, if an individual had a previous RT-PCR/Antigen positive test more than 90 days before the event of interest. Only the first positive test before the current event will be considered. This variable will be created differently for the cohort and TND.
   1. TND: Individuals with a previous RT-PCR or Antigen test positive considering the current sample collection date. (No previously infected, 91-180 days, >180 days)
   2. Cohort: Individuals with a previous RT-PCR or Antigen test considering the study start date (2021/09/15). (No previously infected,28-90 days,91-180 days, >180 days)
2. Interval between second and booster dose

## 4.3 Consistency and error checking

We will check for patterns of missingness and implausible values (e.g. date of second vaccine dose being earlier than the first) for all analytical variables being used, with a record maintained of reasons for excluding any records from analysis. In the case where a variable of interest has high levels of missingness, we will consider using alternative variables that are closely related as a proxy for these missing data.

# Statistical Analyses

### 5.1.1 Exposures of interest

###

Our primary exposure will be receipt of a COVID-19 vaccine booster, with categories to include time since receipt of a booster dose. In this setting, a patient’s vaccination status (exposure) will change over time, as a time-varying exposure.

### 5.1.2 Outcomes of interest

The primary outcome will be a composite outcome of time to COVID-19 hospitalisation or death. COVID-19 hospitalisation will be defined as a RT-PCR confirmed positive test for SARS-CoV-2 in the 14 days prior to admission, or with ICD-10 code for COVID-19 (in any diagnostic position).

COVID-19 deaths will be defined as COVID-19 as the underlying ICD-10 cause of death recorded on the death certificate or death recorded in SIVEP-Gripe^^[[2]](#footnote-1)^^ within 28 days of a confirmed positive test.

Secondary outcomes will be the single outcomes of: i) COVID-19 hospitalisation, ii) COVID-19 deaths, iv) RT-PCR confirmed positive test, provided numbers of events allow. We anticipate the RT-PCR confirmed SARS-CoV-2 infection results to be more susceptible to bias arising from differential ascertainment, and therefore anticipate treating these results as more tentative.

### 5.1.3 Potential confounders

We will consider adjusting for age, sex, socioeconomic position, geography and where possible, comorbidities. More specifically, we anticipate adjusting for the following confounders in Scotland: age (five-year bands^^[[3]](#footnote-2)^^), sex, socioeconomic position (SEP) measured by quintiles of the Scottish Index of Multiple Deprivation (SIMD) (1 refers to most deprived and 5 refers to least deprived), residential settlement measured by the urban/rural 6 fold classification (1 refers to large urban areas and 6 refers to small remote rural areas), household size, number and types of comorbidities commonly associated with COVID-19 illness (asthma, chronic kidney disease, liver cirrhosis, chronic neurological condition, heart failure, diabetes (type 1 and type 2), dementia, coronary heart disease), risk factors (smoking status, blood pressure, body mass index (BMI)) and Health Board.

In Brazil, we anticipate adjusting for age (five-year bands), sex, the primary reason for vaccination, race/ethnicity and calendar week will be accounted for within the statistical models.

### 5.1.4 Potential effect modifiers

Stratification into different population:

- Primary vaccination schedule (2 doses): ChAdOx1, BNT162b2
- Age group (18-49,50-64, 65+ years)
- Period of which variant (Delta or Omicron)
- Additional, given that VE may differ between those with multiple underlying medical conditions, that are related to serious COVID-19, we will stratify analyses by COVID-19 risk group when data allow. We will also consider stratifying analyses by vaccine type and calendar time (to reflect different dominant variants present in the country).

### 5.1.5 Analytical techniques

We will commence analysis by conducting descriptive analyses to visually inspect trends in vaccination delivery, age-specific COVID-19 hospitalisations and COVID-19 deaths, including by age group and sex. This will include checking the number of people who have received two doses and the length of follow-up available following the second dose by vaccine type. Including plotting Lexis diagram to afford for the correlated structure of the data (Calendar Time/Length of Follow-up)

For the analysis consisting of individuals with at least two doses, we will stratify the cohort based on the month of the second dose of each individual. The vaccination status will be classified as:

- Second dose:
  - <2 weeks
  - 2-9 weeks
  - 10-19 weeks
  - ≥20 weeks without a booster dose [Reference Period]
- Booster dose:
  - <2 week
  - 2-4 weeks
  - 5-8 weeks
  - 9-12 weeks
  - ≥13 weeks

This variable will be used to fit a Poisson regression to obtain rate ratios (RRs) and their 95% confidence intervals (CIs). If we observe overdispersion, we will consider using Negative Binomial regression models or Poisson with robust standard errors. Models will be adjusted for the relevant confounders above, including a set of dummy variables for calendar week.

The models will be fit to a dataset with all events and a random sample, without replacement, of 100 individuals per event (for hospitalisation and death, 10 for positive tests). The sample weights will be calculated to represent the sampling fraction, thus ensuring the correct calculation of the person-years at risk for the whole population.

The primary analysis for the test negative will be on individuals who are symptomatic at the time of test and the date of symptom onset is 0-10 days before the test. We will then establish the number of weeks post-vaccination for the cases and controls calculated as time between sample collection date and vaccine dose. The odds' ratio will be calculated using a logistic regression. Adjusted for: race/ethnicity, comorbidities, dummy variables for calendar week, age (five-year bands), sex. Controls will be defined based on a negative test.

We will also construct a propensity score to be tested among those in the primary cohort and use this as an inverse propensity weight.

We will assess vaccine waning looking for statistical evidence of reducing effectiveness, we will do a trend test on the period post-vaccination from 14-27 days onwards. We will also assess an exploratory change point from 28+, 42+, 56+ days to do a linear trend test post-vaccination.

### 5.1.6 Corrections for multiple testing

Following previous epidemiological recommendations, we will not correct for multiple testing [3].

### 5.1.7 Sensitivity analysis

We will consider exploring the impact of alternative approaches to classifying the start of the exposed period (e.g. from day 7 after completion of the vaccination schedule), classifying COVID-19 hospitalisation based on primary diagnosis (rather than any diagnostic position), using only individuals with five days of fewer from symptom onset to test (reducing the risk of false-negative) and statistical adjustment for people with previous COVID-19 infection (rather than excluding them).

### 5.1.8 Other analysis

We will consider conducting falsification analyses (negative controls) for alternative outcomes (e.g. non-COVID hospitalisations) if data allow.

## 5.2 Missing data

Missing data will be reported as percentages of total or raw numbers where possible. For covariates that may have a higher proportion of missing data, we will either use records with no item missingness or use a missing category.

## 5.3 Statistical software

All analyses will be carried out using R/RStudio using the following packages: tidyverse^4^, Epi^5^, survival^6^, mgcv^7^, popEpi^8^

# Reporting results

## 6.1 Reporting guidelines and conventions

Results will be reported according to the Strengthening the Reporting of Observational Studies in Epidemiology (STROBE) and REporting of studies Conducted using Observational Routinely-collected Data (RECORD) guidelines. Measures of the association will be interpreted based on 95% confidence intervals.

## 6.2 Dissemination

This statistical analysis plan will be made publicly available on a website prior to conducting the main statistical analyses. The results will be written in a manuscript and submitted to a peer-reviewed journal. We will also seek to provide feedback to relevant COVID-19 advisory bodies (e.g. UK Joint Committee on Vaccination & Immunisation, Brazilian Ministry of Health, WHO) as appropriate. All code will be made publicly available via a GitHub repository.

# References

[1] US Food and Drug Administration. Development and Licensure of Vaccines to Prevent COVID-19. 2020.

[2] Hodgson SH, Mansatta K, Mallett G, Harris V, Emary KRW, Pollard AJ. What defines an efficacious COVID-19 vaccine? A review of the challenges assessing the clinical efficacy of vaccines against SARS-CoV-2. The Lancet Infectious Diseases. 2021;21(2):e26-e35.

[3] Rothman KJ. No adjustments are needed for multiple comparisons. Epidemiology 1990;1(1):43 46.

[4] Wickham, Hadley, et al. "Welcome to the Tidyverse." *Journal of open source software* 4.43 (2019): 1686.

[5]Carstensen, Bendix, et al. "The Epi Package." (2007).

[6] Therneau, Terry M., and Thomas Lumley. "Package ‘survival’." *Survival analysis Published on CRAN* 2.3 (2014): 119.

[7] Wood, Simon, and Maintainer Simon Wood. "Package ‘mgcv’." *R package version* 1 (2015): 29.

[8] Miettinen, Joonas, Matti Rantanen, and Karri Seppa. "Package ‘popEpi’." (2016).

# Appendix

## **Table S1. ICD-10 codes**

| **Code** | **Description** | **Category** |
| --- | --- | --- |
| U07.1 | COVID-19, virus identified | U07.1 |
| U07.2 | COVID-19, virus not identified | U07.2 |
| Source:<https://www.who.int/classifications/icd/COVID-19-coding-icd10.pdf> | | |

1. Rapid antigen test will be evaluated only in Brazil [↑](#footnote-ref-0)
2. Brazilian data will use only time between test and date of death, while Scotland will use ICD Codes and time between test and date of death and ICD-10 codes in the death record. [↑](#footnote-ref-1)
3. The first band is 7 years: 18-24, the last band is 90+ [↑](#footnote-ref-2)
