# Supplementary material for: Effectiveness of mRNA boosters after homologous primary series with BNT162b2 or ChAdOx1 against symptomatic infection and severe COVID-19 in Brazil and Scotland: A test-negative design case–control study
Source: PLoS Med. 2023 Jan 11;20(1):e1004156. doi: 10.1371/journal.pmed.1004156 (PMC9879484; doi:10.1371/journal.pmed.1004156)
Supplement: Attachment — Submitted filename: cerqueirasilva.pdf [file pmed.1004156.s004.pdf]

This paper presents two cohorts (Brazil and Scotland) of vaccine effectiveness data against SARS–CoV–2 infection and severe COVID–19 outcomes..

I was asked for a statistical report and I interpret that to include all aspects of the design and conduct of the study. The version I received did not have page numbers. My numbering has the abstract starting on page 3

Points of detail

Page 7 We learn that Scotland provided 99% coverage but what about the three Brazilian ones? It is quite hard to find information about the data–bases on–line. For instance, as far as I can tell, the only web presence of SIVEP–Gripe is a GitHub account which seems to be code only.

Page 7 Why are the exposure periods different for the two countries?

Page 7 Why categorise age? Categorising an essentially continuous variable wastes information (Altman and Royston, 2006; Royston et al., 2006) and leads to models which are often implausible as they predict the effect remaining flat within categories and then jumping to a new value at the category boundary.

Page 7 and 8 Presumably not all confounders were used in both countries as Scotland does not have states. Table S1 tells us which region was involved in Brazil but not which state and if that was what was used in the modelling it would be consistent to tell us the breakdown by state.

Page 7 Why are the categories for previous infection different for the two countries?

Page 8 Is the trend test one for linear trend? Is linearity here plausible? How were the values for the predictor arrived at? Equally spaced? Category mid–points?

Page 10 It might be helpful to tell us whether the deprivation categories are based on national cut–offs or are sample specific quintile categories.

Page 11 Table 1 reveals some major differences between the two cohorts. This is hardly surprising but should we not also have some comment somewhere about whether the authors regard this as a good thing or a bad thing for their conclusions.

Page 13 Here we see an isolated result just expressed as a p–value. Apart from adding the usual effect size and confidence interval perhaps tell

us what it refers too? It is not clear, at least to me.

Page 15 I would be very cautious about using Qatar as a comparator. The population there is biased to young ages by the large numbers of single, young male labourers from South Asia many of whom live in communal accommodation.

Page 16 'largest to our knowledge' would be safer.

Point of more substance

I am not entirely clear what overall message the authors wish to draw from having two cohorts. This makes it difficult to evaluate the appropriateness of the statistical methods used. With such large sample sizes any difference which might have a substantive importance is bound to achieve some arbitrary level of statistical significance and the authors appear to have refrained from making any formal comparisons between Brazil and Scotland, probably wisely. On the other hand if the authors' contention is that the two cohorts are essentially similar in their vaccine response then that does not seem to be expressed and indeed does not seem to be true.

Summary

Apart from a number of points for clarification we need some explicit statement of what message we are being encouraged to draw.

Michael Dewey

References

D G Altman and P Royston. The cost of dichotomising continuous variables. *British Medical Journal*, 332:1080, 2006.

P Royston, D G Altman, and W Sauerbrei. Dichotomizing continuous predictors in multiple regression: a bad idea. *Statistics in Medicine*, 25:127–141, 2006.
