# Supplementary material for: Effectiveness of mRNA boosters after homologous primary series with BNT162b2 or ChAdOx1 against symptomatic infection and severe COVID-19 in Brazil and Scotland: A test-negative design case–control study
Source: PLoS Med. 2023 Jan 11;20(1):e1004156. doi: 10.1371/journal.pmed.1004156 (PMC9879484; doi:10.1371/journal.pmed.1004156)
Supplement: Attachment — Submitted filename: cerqueirasilva.pdf [file pmed.1004156.s006.pdf]

This paper presents two cohorts (Brazil and Scotland) of vaccine effectiveness data against SARS–CoV–2 infection and severe COVID–19 outcomes..

I was asked for a statistical report and I interpret that to include all aspects of the design and conduct of the study. When I am asked to look at a re–submission which I had already reviewed on a previous submission I restrict myself on principle to commenting on how the authors have responded to my original comments. I do not review the article again from scratch unless it has been so changed as to amount effectively to a new submission.

Points of detail

I said:

> *We learn that Scotland provided 99% coverage but what about the three Brazilian ones? It is quite hard to find information about the data–bases on–line. For instance, as far as I can tell, the only web presence of SIVEP–Gripe is a GitHub account which seems to be code only.*

The authors have provided more detail but we do not seem to have an equivalent figure to the 99% quoted for Scotland.

I said:

> *Why categorise age? Categorising an essentially continuous variable wastes information (Altman and Royston, 2006; Royston et al., 2006) and leads to models which are often implausible as they predict the effect remaining flat within categories and then jumping to a new value at the category boundary.*

The authors seem to have assumed I was intending to use a linear model for age here when I suggested continuous. There are many more suitable choices like fractional polynomials, splines, or even polynomials although the latter have their drawbacks too (Magee, 1998). However given the resulta of the linear fit this may be unnecessary.

I said:

> *I am not entirely clear what overall message the authors wish to draw from having two cohorts. This makes it difficult to evaluate the appropriateness of the statistical methods used. With such large sample sizes any difference which might have a substantive importance is bound to achieve some arbitrary level of statistical significance and the authors appear to have refrained from making*

> *any formal comparisons between Brazil and Scotland, probably wisely. On the other hand if the authors' contention is that the two cohorts are essentially similar in their vaccine response then that does not seem to be expressed and indeed does not seem to be true.*

I was not suggesting merging the two cohorts, or indeed any other analysis option. In fact I clearly stated that I thought the authors had been wise not to do that¿ What I meant was that it is not clear to me what scientific question is being answered by having the two cohorts in the same article. Are the authors claiming that their findings gather strength from the similarities between the cohorts despite Brazil and Scotland being quite different countries, or are they pointing to important differences which may be informative, or is this really two separate papers?

Michael Dewey

References

D G Altman and P Royston. The cost of dichotomising continuous variables. *British Medical Journal*, 332:1080, 2006.

L Magee. Nonlocal behavior in polynomial regressions. *American Statistician*, 52:20–22, 1998.

P Royston, D G Altman, and W Sauerbrei. Dichotomizing continuous predictors in multiple regression: a bad idea. *Statistics in Medicine*, 25:127–141, 2006.
