# Supplementary material for: Effectiveness of mRNA boosters after homologous primary series with BNT162b2 or ChAdOx1 against symptomatic infection and severe COVID-19 in Brazil and Scotland: A test-negative design case–control study
Source: PLoS Med. 2023 Jan 11;20(1):e1004156. doi: 10.1371/journal.pmed.1004156 (PMC9879484; doi:10.1371/journal.pmed.1004156)
Supplement: Attachment — Submitted filename: Response Letter.docx [file pmed.1004156.s005.docx]

# Editor points

## 1.

Existing literature (see Effectiveness of COVID-19 vaccines against SARS-CoV-2 variants of concern: a systematic review and meta-analysis. Zeng B, Gao L, Zhou Q, Yu K, Sun F. BMC Med. 2022 May 23;20(1):200) has shown that ChAdOx1 does not perform the same as BNT162b2. Due to potentially introducing confounding by primary vaccination series, please do not group these two together and compare primary vaccination series of BNT162b2+mRNA booster and primary vaccination series of ChAdOx1+mRNA booster with unvaccinated. On the same note in lines 292-307, please contextualize discussion of other studies by the primary vaccination series + booster in each country/study rather than exclusively grouping by number of vaccination doses.

**A: We thank the reviewer for the comment. We have provided separate data for ChAdOx1 and BNT162b2**

## 2.

The 13 week threshold used is unorthodox and inconsistent with other studies. As suggested by one of the reviewers, agree extending out to 6 months and evaluating greater than or equal to 6 months as the last waning threshold makes sense. If this is not possible at least use greater than or equal to 3 months (or 12 weeks).

**A: We have updated the analysis stratifying by month after the booster dose (0-13, 14-29 days, 1 month, 2 months, 3 months and greater or equal to 4 months after booster dose)**

## 3.

Given the benefits of a fourth dose for specific populations (HCWs, elderly, immunocompromised (<https://www.who.int/news/item/17-05-2022-interim-statement-on-the-use-of-additional-booster-doses-of-emergency-use-listed-mrna-vaccines-against-covid-19>). Would suggest the authors consider updating the analysis to also include a second booster dose for whatever periods of follow-up and populations are available.

**A**: **We have updated the analysis to the newer database available (end of April). However, unfortunately, we only have sufficient numbers to estimate the vaccine effectiveness of a second booster dose against symptomatic infection in Scotland. Those estimates were included in the revised manuscript**

## 4.

The Qatari study has now been published and can be updated. Chemaitelly H, Ayoub HH, Al Mukdad S,et al. Duration of mRNA vaccine protection against SARS-CoV-2 Omicron BA.1 and BA.2 subvariants in Qatar. Nat Commun. 2022 June 2;13(1):3082.

**A: We have updated all references of a preprint to the published version**

## 5.

In the limitations discussion sublineages can also add BA4 and BA 5 now

**A: We have included the limitation of the analysis with non-distinct omicron sublineages (including BA4/5) and updated the following sentence to reflect the actual scenario:
“**Third, we were unable to discriminate between Omicron lineages in our study. Nevertheless, initial studies have suggested VE against omicron subvariants to be similar.[8,28,29]”

# 6.

Title: We suggest revising the title for clarification. Please revise your title according to PLOS Medicine's style. Your title must be nondeclarative and not a question. It should begin with the main concept if possible. "Effect of" should be used only if causality can be inferred, i.e., for an RCT. Please place the study design ("A randomized controlled trial," "A retrospective study," "A modelling study," etc.) in the subtitle (ie, after a colon).

**A: We have revised the title according to PLOS medicine's style: "Effectiveness of mRNA boosters after homologous primary series with BNT162b2 or ChAdOx1 against symptomatic infection and severe COVID-19 in Brazil and Scotland: a test-negative design case-control study.**"

## 7.

Data availability statement: Please provide the weblink/email contact information for requests to access the data from Scotland.

**A: We have added a section with information regarding data availability, as follows:**

**"Data availability
One of the study coordinators (M.B.-N.) signed a term of responsibility on using each database made available by the Ministry of Health (MoH). Each member of the research team signed a term of confidentiality before accessing the data. Databases were manipulated in a secure computing environment, ensuring protection against data leakage. The Brazilian National Commission in Research Ethics approved the research protocol (CONEP approval no. 4.921.308). Our agreement with the MoH for accessing the databases patently denies authorization of access to a third party. Any information for assessing the databases must be addressed to the Brazilian MoH at** [**https://datasus.saude.gov.br/**](https://datasus.saude.gov.br/)**, and requests can be addressed to datasus@saude.gov.br. In this study, we used anonymized secondary data following the Brazilian Personal Data Protection General Law, but it is vulnerable to re-identification by third parties as they contain dates of relevant health events regarding the same person. To protect the research participants' privacy, the approved Research Protocol (CONEP approval no. 4.921.308) authorizes the dissemination only of aggregated data, such as the data presented here.For Scotland, data dictionary covering the datasets used in this study can be found at https://github.com/EAVE-II/EAVE-II-data-dictionary. The data used in this study are sensitive and will not be made publicly available."**

## 8.

Abstract: Please structure your abstract using the three PLOS Medicine headings (Background, Methods and Findings, Conclusions). Please combine the Methods and Findings sections into one section, "Methods and findings".

**A: We have modified the headings and mixed Methods and Findings in the abstract.**

## 9.

Abstract: Methods and Findings: Please describe the national databases, in terms of the populations represented and numbers of participants included, as well as the time period of the study, the maximum length of follow up, and how the main outcome measures were defined/quantified. We suggest including information similar to what is reported at lines 115-117 of the Methods ("All individuals aged 18 years or older who reported acute respiratory illness symptoms and tested for SARS-CoV-2 infection between January 1, 2022 and March 7, 2022 for Brazil and December 20, 2021 and March 9, 2022 for Scotland were eligible for the study").

**A: We included the sentence: "The inclusion criteria were all individuals aged 18 years or older who reported acute respiratory illness symptoms and tested for SARS-CoV-2 infection between January 1, 2022 and April 23, 2022 for Brazil and Scotland."**

## 10.

Abstract: Methods and Findings: Please report p values as p<0.001 where applicable.

**A: We only have provided point estimates and 95% Confidence interval for all estimates.**

## 11.

**Abstract: Methods and Findings: Please include a summary of adverse events if these were assessed in the study.**

**A: Adverse events were not assessed in the study.**

## 12.

Abstract: Methods and Findings: In the last sentence of the Abstract Methods and Findings section, please describe the main limitation(s) of the study's methodology.

**A: We included the sentence: " One limitation of this study was that we assumed that all cases included in the analysis were due to the Omicron variant based on the period of dominance."**

## 13.

Abstract: Conclusions: Please address the study implications without overreaching what can be concluded from the data; the phrase "In this study, we observed ..." may be useful.

**A: Conclusions were based on study results: " We observed that mRNA boosters after a primary vaccination course with either mRNA or viral-vector vaccines provided modest, short-lived protection against symptomatic infection with Omicron but substantial and more sustained protection against severe COVID-19 outcomes for at least 3 months."**

## 14.

Author summary: At this stage, we ask that you include a short, non-technical Author Summary of your research to make findings accessible to a wide audience that includes both scientists and non-scientists. The Author Summary should immediately follow the Abstract in your revised manuscript. This text is subject to editorial change and should be distinct from the scientific abstract. Please see our author guidelines for more information:<https://journals.plos.org/plosmedicine/s/revising-your-manuscript#loc-author-summary>

**A: We included the author summary in the MS**

## 15.

Main text: Please place in-text citations of references within brackets, before the sentence punctuation.

**A: We have updated all the references to the aforementioned format.**

## 16.

Methods: For all observational studies, in the manuscript text, please indicate: (1) the specific hypotheses you intended to test, (2) the analytical methods by which you planned to test them, (3) the analyses you actually performed, and (4) when reported analyses differ from those that were planned, transparent explanations for differences that affect the reliability of the study's results. If a reported analysis was performed based on an interesting but unanticipated pattern in the data, please be clear that the analysis was data-driven.

**A: we have included the planned analysis as an appendix and one paragraph regarding an unplanned analysis in the methods section.**

## 17.

Methods: Line 145: Please explain "comorbidities" and please describe all factors that were adjusted for, and explain how these data were obtained.

**A: We have included this information in the methods section**

“number of medical comorbidities (Brazil-diabetes mellitus, obesity, immunosuppression, chronic respiratory disease, cardiac disease and chronic kidney disease) or QCovid risk group (Scotland), state of residence (Brazil) or geographic area (Scotland), and previous infection (none, 3-5 months ago, 6-12 months and > 1 year), calendar time was included as the week of sample collection, and healthcare worker in Brazil and number of previous RT-PCR in Scotland (as a proxy for healthcare worker). In Scotland, we additionally adjusted for the residential settlement type (six categories, from large urban to small remote rural areas) and household size. QCovid risk groups are characteristics used in the QCOVID algorithm to predict the risk of hospital admission and death due to COVID-19[15].”

## 18.

Methods: Line 151: Please describe QCovid risk group here.

**A: We have provided a brief description of QCovid risk. “QCovid risk groups are characteristics used in the QCOVID algorithm to predict the risk of hospital admission and death due to COVID-19.[15]”**

## 19.

Methods: Line 165-166: Please clarify if this refers to weeks: "A trend test, evaluating the change in estimate between 2-4, 5-8, 9-12, and ≥13 past booster dose…"

**A: The sentence was modified, as informed in the answer of Q2, and the period was clarified: "In both countries, the exposure periods were unvaccinated, first dose (0-13 days, 14 days to 1 month, 2 to 5 months, ≥6 months ), second dose (0-13 days, 14 days to 1 month, 2 to 4 months, ≥ 5 months), and booster dose (0-13 days, 14 to 29 days, 1 month, 2 months, 3 months and ≥ 4 months)." We removed the trend test as it does not add extra information to the interpretation.**

## 20.

Methods: Statistical analyses: For analyses where a p value is given, please specify the statistical test used to determine it as well as the significance level used (eg, P<0.05, two-sided).

**A: We only have provided point estimates and 95% Confidence interval for all estimates.**

## 21.

Methods: Please ensure that the study is reported according to the STROBE guideline. Thank you for including the completed STROBE checklist as Supporting information.

Please add the following statement, or similar, to the Methods: "This study is reported as per the Strengthening the Reporting of Observational Studies in Epidemiology (STROBE) guideline (S1 Checklist)."

When completing the checklist, please use section and paragraph numbers throughout to indicate locations within the text.

**A: We have included the sentence in the methods and updated the strobe checklist to paragraph numbers.**

## 22.

Methods: Did your study have a prospective protocol or analysis plan? Please state this (either way) early in the Methods section.

a) If a prospective analysis plan (from your funding proposal, IRB or other ethics committee submission, study protocol, or other planning document written before analyzing the data) was used in designing the study, please include the relevant prospectively written document with your revised manuscript as a Supporting Information file to be published alongside your study, and cite it in the Methods section. A legend for this file should be included at the end of your manuscript.

b) If no such document exists, please make sure that the Methods section transparently describes when analyses were planned, and when/why any data-driven changes to analyses took place.

c) In either case, changes in the analysis-- including those made in response to peer review comments-- should be identified as such in the Methods section of the paper, with rationale.

**A: We have a statistical analysis plan. We included it as a supplementary file. However, the methods were changed to answer the reviewers's requests.**

## 23.

Results: Line 238-248: Please also provide p values for these results, where applicable.

**A: We now only provide point estimates and their confidence intervals for all estimates.**

## 24.

Discussion: Please present and organize the Discussion as follows: a short, clear summary of the article's findings; what the study adds to existing research and where and why the results may differ from previous research; strengths and limitations of the study; implications and next steps for research, clinical practice, and/or public policy; one-paragraph conclusion.

**A: The Discussion section was organized as suggested.**

## 25.

Discussion: Line 267: Please clarify "severe Omicron COVID-19 outcomes" to indicate that this means Omicron era cases.

**A: We have changed the sentence to clarify the message.
"severe COVID-19 cases during the predominance of Omicron variant for at least 3 months"**

## 26.

Figures and Tables: Please provide titles and legends for all figures (including those in Supporting Information files). Please check that any abbreviations used within Tables and Figures is completely defined in the legend. Please consider avoiding the use of red and green together in the same figure in order to make your figure more accessible to those with color blindness.

**A: We have edited the colours of figures to colourblind friendly**

## 27.

Figure 1 and Figure 2: Please indicate in the figure caption the meaning of the error bars. Please indicate in the legend if severe COVID-19 indicates both hospitalization and death.

**A: We have now included the requested information about the error bars and the definition of severe COVID-19 in the legend.**

## 28.

S1 Table: Please describe in the Methods how characteristics of cases and controls were evaluated/reported. Please describe how characteristics (e.g. obesity/immunosuppression/respiratory disease/kidney disease/cardiac disease etc) were determined. How was race/ethnicity defined and by whom? In S1 part B, please use variable names under "characteristic" that are more intuitive and/or similar to S1 part A.

**A: All data used in our analysis came from structured administrative databases. No detailed information about the definition of the characteristics was provided.**

**We have updated table S1B to provide more intuitive names.**

## 29.

S2 Table: Please note all values are n(%).

**A: We have included a legend for all tables indicating the n(%)**

## 30.

S3 Table: Please include at the top that the categories represent ages. In Part B (Scotland, severe outcomes): In the legend please define cells with "*" indicated.

**A: We have included the definition of * for all tables.**

## 31.

S4 Table, S5 Table: Where p values are reported, please report as p<0.001 where applicable. When p<0.01 please report to 3 decimal places, and when p>/=.01 please report to two decimal places. Please indicate that the table is presented by age category. In the legend, please define cells with "-" reported.

**A: We now only provide point estimates and their confidence intervals for all estimates.**

## 32.

S6 Table: Please label the columns at the top with "age" and "primary series" as appropriate. In the legend for Part B, please define the cells with "*" reported.

**A: We have included the definition of * for all tables.**

## 33.

S1 Appendix: There seems to be an unlabelled table between S6 Table and S7 Table, titled "Severe Outcomes" and reporting VE for each primary series type.

**A: We have fixed all the inconsistencies in the supplementary tables.**

## 37.

S7 Table: Part A: The right-hand side of the table is cut off.

**A: We have fixed all the inconsistencies in the supplementary tables.**

# REVIEWER 1

This paper presents two cohorts (Brazil and Scotland) of vaccine e_ectiveness data against SARS{CoV{2 infection and severe COVID{19 outcomes.. I was asked for a statistical report and I interpret that to include all aspects of the design and conduct of the study. The version I received did not have page numbers. My numbering has the abstract starting on page 3

## 1.

Page 7 We learn that Scotland provided 99% coverage but what about the three Brazilian ones? It is quite hard to find information about the databases on-line. For instance, as far as I can tell, the only web presence of SIVEP-Gripe is a GitHub account which seems to be code only.

**A: We have now included more information about Brazilian databases. The SIVEP-Gripe is a surveillance database for severe acute respiratory syndrome. The system was created in 2009 during the H1N1 pandemic; all hospitalized cases suspected of SARS-CoV-2 are reported in this database. The e-SUS is a surveillance system for acute respiratory illness and requires mandatory notifications since 2020.**

**“**The data in Brazil came from three deterministically linked national datasets provided by the Ministry of Health: COVID-19 Vaccination Campaign (SI-PNI); Acute Respiratory Infection Suspected Cases (e-SUS-Notifica); and Severe Acute Respiratory Infection/Illness (SIVEP-Gripe). All COVID-19 vaccine doses in Brazil are free of charge by the Ministry of Health, while all suspected and confirmed cases of COVID-19 must be notified in the e-SUS-Notifica or SIVEP surveillance systems[2].”

## 2.

Page 7 Why are the exposure periods different for the two countries?

**A: We have now updated the analysis to harmonize both exposure periods in the two countries.**

## 3.

Why categorize age? Categorizing an essentially continuous variable

wastes information (Altman and Royston, 2006; Royston et al., 2006) and leads to models which are often implausible as they predict the effect remaining at within categories and then jumping to a new value at the category boundary.

**A: Age as continuous is only valid if one can be confident that the risk monotonically increasing (or decreasing) by age. Otherwise, fitting age as a continuous variable will result in a reduced control of confounding by this variable. Thus, given that five-year age-bands are small, and therefore that residual confounding is unlikely, and that we have very large sample sizes (so there is no problem with lack of degrees of freedom), we prefer to use age-categories. However, as a sensitivity analysis, we have run the model using age as a continuous variable. Little or no difference was observed compared to categorizing age (Table below).**

| ChAdOx1 | | BNT16b2 | |
| --- | --- | --- | --- |
| Age (Five year band) | Age (continuous) | Age (Five year band) | Age (continuous) |
| 88.1 (87.1 — 89.1) | 89.4 (88.5 — 90.3) | 92.8 (91.0 — 94.2) | 93 (91.3 — 94.4) |
| 93.5 (93.0 — 94.0) | 94.2 (93.7 — 94.7) | 92.7 (91.0 — 94.0) | 92.9 (91.2 — 94.2) |
| 91.9 (91.4 — 92.3) | 92.3 (91.8 — 92.7) | 92.8 (91.1 — 94.1) | 92.9 (91.2 — 94.2) |
| 88.3 (87.5 — 89.0) | 87.8 (87.0 — 88.5) | 89 (84.4 — 92.3) | 89 (84.3 — 92.3) |
| 86.2 (84.9 — 87.4) | 84.8 (83.4 — 86.1) | 86.6 (72.3 — 93.5) | 86.5 (72.1 — 93.5) |
| 82.3 (79.7 — 84.7) | 79.4 (76.3 — 82.1) | 74.1 (9.1 — 92.6) | 73.6 (7.6 — 92.5) |

## 4.

Page 7 and 8 Presumably not all confounders were used in both countries as Scotland does not have states. Table S1 tells us which region was involved in Brazil but not which state and if that was what was used in the modelling it would be consistent to tell us the breakdown by state.

**A: We updated Table S1 to provide more information including the numbers by state.**

## 5.

Page 7 Why are the categories for previous infection different for the two countries?

**A: We have harmonized the analysis, and the previous infection categories are the same in both countries: none, 3-5 months ago, 6-12 months and > 1 year**

## 8.

Page 8 Is the trend test one for linear trend? Is linearity here plausible?

How were the values for the predictor arrived at? Equally spaced?

Category mid-points?

**A**: **The trend test is a linear contrast using the regression coefficients of the group past 14 days of the booster dose. To test if there is a linear relationship of decreasing or increasing between the levels. We removed the trend test as it does not add extra information to the interpretation.**

## 9.

Page 10 It might be helpful to tell us whether the deprivation categories are based on national cut-offs or are sample specific quintile categories.

**A: Both deprivation quintiles are based on national cut-offs. (We updated the methods section to contains this information)**

**“**socioeconomic position (Brazil: Brazilian Municipality Deprivation Index; Scotland: Scottish Index of Multiple Deprivation, both deprivation indexes used national cut-offs to define the quintiles),”

## 10.

Page 11 Table 1 reveals some major differences between the two cohorts.

This is hardly surprising but should we not also have some comment somewhere about whether the authors regard this as a good thing or a bad thing for their conclusions.

**A: We have now acknowledged this limitation in the discussion.**

## 11.

Page 13 Here we see an isolated result just expressed as a p-value. Apart from adding the usual effect size and confidence interval perhaps tell us what it refers too? It is not clear, at least to me.

**A: The p-value was related to the trend-test (the effect estimate and confidence interval was presented in the supplementary table). We removed the trend test as it does not add extra information to the interpretation.**

## 12.

Page 15 I would be very cautious about using Qatar as a comparator. The population there is biased to young ages by the large numbers of single, young male labourers from South Asia many of whom live in communal accommodation.

**A: We agree with the reviewer about the suggestion of being cautious in comparing our results with the Qatar ones. However, the Qatar study population from that article is well-balanced in terms of sex, despite being very biased to a younger age.**

## 13.

Page 16 `largest to our knowledge' would be safer.

**A: We thank the reviewer for the suggestion. We modified the sentence as suggested.**

## 14.

I am not entirely clear what overall message the authors wish to draw from having two cohorts. This makes it difficult to evaluate the appropriateness of the statistical methods used. With such large sample sizes any difference which might have a substantive importance is bound to achieve some arbitrary level of statistical significance and the authors appear to have refrained from making any formal comparisons between Brazil and Scotland, probably wisely. On the other hand if the authors' contention is that the two cohorts are essentially similar in their vaccine response then that does not seem to be expressed and indeed does not seem to be true.

Apart from a number of points for clarification we need some explicit statement of what message we are being encouraged to draw.

**A: We have considered each country a separate cohort due to the different structural characteristics of each country, which could lead to different biases. Due to the large sample size in Brazil compared to Scotland, if the analysis were conducted as one cohort, any potential bias arising from the Brazilian study will overtake the ones arising from Scottish data. The separated approach allows us to extract more robust findings from the question evaluated.**

# Reviewer 2

Review Cerqueira-Silva et al, PlosMed
The strength of this study is that it takes place in two countries, with different populations but similar vaccination strategies, among large populations.  The design is solid and well-execulted.   I have two high-level critiques.  First, is that they call this a study of waning, even in the title, but only follow-up persons to 13+ weeks--- not sure exactly how long in that furthest follow-up group.  This relatively short follow-up time is sufficient for symptomatic infection with omicron where waning happens swiftly, but probably not enough followup time for severe disease.  2.  The authors overstate the amount of waning after booster among persons >=65 years.   Their results show a minimal amount of decrease in VE (<10 percentage points) with overlapping CI< yet they lead off their discussion by highlighting the waning in this group.

A: **We have increased the follow-up period and modified the conclusions regarding the waning in the elderly as described below.**

## 1.

Line 94.  Delete « offered »

**A: We thank the reviewer for noticing this typo.**

## 2.

Line 112.  Lateral flow tests have lower sensitivity. Most tests in Brazil were lateral flow (Table 1)  Authors could mention this as a potential limitation, due to misclassification of case status.  Do they have test characteristics for the lateral flow tests used?

**A: Unfortunately, we don't have data about the different diagnostics kits used in Brazil. We have included this as a potential limitation. Thanks for suggesting this.**

“Second, most of the tests performed in Brazil during the study period were lateral flow, which may induce bias in the results due to misclassifying cases as controls.”

## 3.

Line 142.  What is vaccination coverage in general populations?   If the unvaccinated percentage is small (<10%)  it could suggest a potential bias among the remaining unvaccinated population in terms of their risk of exposure.  From table 1, it seems that this is <10%, so this should be mentioned as a potential bias in the discussion (if not already).

**A: Indeed, the percentage of unvaccinated people in both countries is close to 10% and the behaviour of those individuals can be very different from vaccinated ones. However, we performed the sensitivity analysis using only vaccinated individuals (Booster dose vs non-booster dose), and the pattern of the results stay similar**

## 4.

Line 158.  Given the matched enrollment in Scotland, how were the matched groups identified in the analysis.   Why not conditional logistic regression?

**A: We have now used unmatched designs in both countries.**

## 5.

Line 166.  Missing "weeks"?

**A: Thanks for noticing the missing word. As we updated the databases and added more follow-up time, we performed the analysis by months instead of weeks. The text was changed thoroughly to reflect this update.**

## 6.

Line 170.  Note that this sensitivity analysis will calculate a relative VE rather than an absolute VE.  If there is still residual VE from two doses, then the relative Ve will be lower than absolute VE.   More an issue for severe disease. it should be stated that this analysis yields a relative VE.   This is seen in table S7 especially in Scotland.

**A: We have now clarified that it is relative VE across all manuscripts.**

## 7.

Line 186.  Replace word "and" with "compared with"??

**A: Thanks for the suggestion. We have changed the sentence.**

## 8.

Line 188.  The median follow-up time since vaccination / booster should be given.  In the >13 week group, what is the median folllowup time in that group.  If it is not much more than 13 weeks, it is not very long.

Table 1.  somewhat surprising that there are a greater percentage in the highest deprivation index categories in Scotland than Brazil.  Why?  Use of different deprivation indices?

**A: We have now included the median and interval interquartile information for each vaccination status as a supplementary table. The longest follow-up now is >4 months (with 130 days of median time). The difference in deprivation percentages is because we have used the municipality deprivation index in Brazil, which can over-represent the largest cities, we have included a sentence discussing this issue in the discussion section.
“**Sixth, there are a disproportional number of individuals in our sample from Brazil’s first quintile of deprivation (low deprivation). It is likely due to the use of the municipality deprivation index, indicating that cities with lower deprivation maintain a broader testing policy than cities with higher deprivation”

**We included supplementary table S3 with median and interval interquartile of each period and vaccine type.**

## 9.

Line 220, figures.  The VE in Brazil was consistently higher, including by age group.   Why do the authors think this is the case?  They should comment on that.   Although table 1 shows low prior infection in both countries, one wonders whether seroprevalence in the Brazilian population was higher, and most people didn't know, which would have lead to more hybrid immunity in that population.

**A: Thanks for raising this point. We have included this topic in the limitation paragraph.**

## 10.

Line 268.   I don't think the data supports this statement that there is significant waning against severe disease in the >65 yo group.  Figure 2 does show some drop in percentage point VE, but not lower than 80% and with overlapping confidence intervals.  The authors state on line 227:  modest waning from 89.5% (95%CI 88.0 - 90.8) at 2-4 weeks to 85.1% (95%CI 83.5 - 86.6) at 13 weeks or more.  This is only 4 percentage points drop.   this statement should be modified lest it be misinterpreted as these vaccines are not working in this high risk age group.

Line 293 and whole paragraph.  Same comment about overstating of waning against severe disease in the elderly.

**A: We have downtone all manuscript regarding to the presence of waning against severe outcomes**

## 11.

Line 315.  There is no evidence that VE differs by BA1 and BA2 - data from UK, Qatar.  No date on other omicron lineages.   Probably not the same amount of difference in VE as with different variants in circulation.

**A: We thank the reviewer for this information, we have updated the paragraph of limitation to reflect this:**

**"Third, we were unable to discriminate between Omicron lineages (BA.1, BA.2, BA.3, BA.4 or BA.5) in our study. Nevertheless, initial studies have suggested VE against omicron subvariants to be similar.[8,30]"**

## 12.

Line 333.  This does not seem to be the case for this vaccine.  You could delete this limitation.

Instead I think a significant limitation to mention is the limited amount of time of follow-up post booster, which limits the assessment of waning.

**A: We removed the statement about the unexpected effects of COVID-19 vaccine in other acute respiratory illness. Additionally, we have added the sentence: "The durability of protection against hospitalization and death remains an open question underscoring the need for studies with extended follow-up periods."**

## 13.

Table S3.  The negative VE >20 weeks after primary series in Scotland should be discussed.  This is not a plausible finding and suggests  a bias among those first vaccinated.  Moreover, this seems more of an issue among the older age groups, and also evident to a lesser degree after booster.  Most other studies that initially showed a negative VE found that it was no longer negative after adding more omicron cases.  Scotland is an outlier in not having the strongly negative VE disappear.

**A: After updating the database to April 23 and the analysis procedures, all strongly negative VE estimates disappear in Scotland. In Brazil, we only observe some small negative estimates in the youngest group (18-49 years). We have included an exploratory analysis to investigate the likely cause and discussed the findings in the manuscript.**

## 14.

Table S4.  Trend test for what?  Not clear.

**A: The trend test is a linear contrast using the regression coefficients of the group past 14 days of the booster dose. To test if there is a linear relationship of decreasing or increasing between the levels. We removed the trend test as it does not add extra information to the interpretation.**

# Reviewer #3:

This is a valuable and important paper that addresses clinical efficacy of mRNA vaccine boosting after mRNA or ChAdOx1 primary series vaccination. The comparison of two countries, with broadly comparable results, gives confidence.

## 1.

This is a comparison to unvaccinated subjects. The recommendation from the paper is that booster vaccination should be promoted. How do the findings compare to people who have primary series vaccination but no booster?

**A: The booster doses provided additional protection, even when compared with individuals with two doses (primary series). Ranging from 50 to 80% of additional protection compared to individuals with ChAdOx1 vaccination and 40 to 60% in those with BNT162b2**

## 2.

Table 1 shows that the demographics of the case positive and use negative groups are somewhat dissimilar. Have these been assessed for statistical difference, and could this influence the analysis.

**A: All the variables presented in Table 1 were included as covariates in the logistic model. As detailed in the methods section: "we adjusted for the following potential confounders: age (five-year bands), sex, socioeconomic position (Brazil: Brazilian Municipality Deprivation Index; Scotland: Scottish Index of Multiple Deprivation, in both deprivation index were used national cut-offs to define the quintiles), number of comorbidities (Brazil) or QCovid risk group (Scotland), state of residence (Brazil) or geographic area (Scotland), and previous infection (none, 3-5 months ago, 6-12 months and > 1 year), calendar time was included as the week of sample collection, and healthcare worker in Brazil and number of previous RT-PCR in Scotland (as proxy for healthcare worker). In Scotland, we additionally adjusted for the residential settlement type (six categories, from large urban areas to small remote rural areas), and household size (Appendix pg 4-6)"**

## 3.

Considerable emphasis is given to waning in subjects >65 years in Brazil whereas the values are not markedly dissimilar from the total cohort.  Is there any reason why this was picked out ?

**A: The focus was on individuals with >65 years because of the presence of statistical evidence of waning against severe outcomes.**
