# Supplementary material for: Effectiveness of mRNA boosters after homologous primary series with BNT162b2 or ChAdOx1 against symptomatic infection and severe COVID-19 in Brazil and Scotland: A test-negative design case–control study
Source: PLoS Med. 2023 Jan 11;20(1):e1004156. doi: 10.1371/journal.pmed.1004156 (PMC9879484; doi:10.1371/journal.pmed.1004156)
Supplement: Attachment — Submitted filename: Revision 2 - PMED.docx [file pmed.1004156.s007.docx]

**Reviewer 1**
This paper presents two cohorts (Brazil and Scotland) of vaccine effectiveness data against SARS–CoV–2 infection and severe COVID–19 outcomes.

I was asked for a statistical report and I interpret that to include all aspects of the design and conduct of the study. When I am asked to look at a re– submission which I had already reviewed on a previous submission I restrict myself on principle to commenting on how the authors have responded to my original comments. I do not review the article again from scratch unless it has been so changed as to amount effectively to a new submission.

**Points of detail**

I said:

*We learn that Scotland provided 99% coverage but what about the three Brazilian ones? It is quite hard to find information about the data–bases on–line. For instance, as far as I can tell, the only web presence of SIVEP–Gripe is a GitHub account which seems to be code only.*

The authors have provided more detail but we do not seem to have an equivalent figure to the 99% quoted for Scotland.

**A: We have updated the text in the methods section. All SARS cases in Brazil, regardless of aetiology, must be notified in the SIVEP-Gripe, and all COVID-19 vaccine doses were provided by the Ministry of Health, so both datasets should cover 100% of the population. The mild cases (e-SUS notifica) are also compulsory registered but those individuals who do not seek a health care facility will not be investigated, therefore will not appear in the system.**

“All COVID-19 vaccine doses in Brazil are provided free of charge by the Ministry of Health. All suspected and confirmed cases of COVID-19 must be notified in the e-SUS-Notifica. All severe acute respiratory illness cases, regardless of aetiology, must be notified in the SIVEP surveillance system. Therefore, these three datasets should provide 100% country-wide coverage of all notified cases [15].”

I said:

*Why categorise age? Categorising an essentially continuous vari- able wastes information (Altman and Royston, 2006; Royston et al., 2006) and leads to models which are often implausible as they predict the effect remaining flat within categories and then jumping to a new value at the category boundary.*

The authors seem to have assumed I was intending to use a linear model for age here when I suggested continuous. There are many more suitable choices like fractional polynomials, splines, or even polynomials although the latter have their drawbacks too (Magee, 1998). However given the result of the linear fit this may be unnecessary.

**A: We are sorry about our misinterpretation. We agree with the reviewer that splines or fractional polynomials should provide a better fit for continuous variables. In our case, as pointed out by the reviewer, fitting using a more flexible function will change the coefficients by only a very minimal amount, so that we will stick with the results from the binned age model.**

I said:

*I am not entirely clear what overall message the authors wish to draw from having two cohorts. This makes it difficult to evaluate the appropriateness of the statistical methods used. With such large sample sizes any difference which might have a substantive importance is bound to achieve some arbitrary level of statistical significance and the authors appear to have refrained from making any formal comparisons between Brazil and Scotland, probably wisely. On the other hand if the authors’ contention is that the two cohorts are essentially similar in their vaccine response then that does not seem to be expressed and indeed does not seem to be true.*

I was not suggesting merging the two cohorts, or indeed any other analysis option. In fact I clearly stated that I thought the authors had been wise not to do that¿ What I meant was that it is not clear to me what scientific question is being answered by having the two cohorts in the same article. Are the authors claiming that their findings gather strength from the similarities between the cohorts despite Brazil and Scotland being quite different countries, or are they pointing to important differences which may be informative, or is this really two separate papers?

**A: Our approach to conducting harmonized analysis in two different countries allows us for indirect validation of the results. Under the assumption that confounding structures differ between the two countries, similar findings allow us to draw reliable conclusions. [1]**

1 - Lawlor, Debbie A., Kate Tilling, and George Davey Smith. "Triangulation in aetiological epidemiology." *International journal of epidemiology* 45.6 (2016): 1866-1886.

**Requests from the editors:**

GENERAL

Thank you for addressing previous reviewer and editor comments

Please address all editor and reviewer comments detailed below and in the attached file

DATA AVAILBILITY STATEMENT

Thank you for updating your data availability statement. Please also include this in your manuscript submission form – the older statement is still placed here – and remove from the end of the manuscript.

**A: We have removed the data availability statement from the end of the manuscript and included it in the submission form only.**

ABSTRACT

Please quantify main results with p-values as well as 95% CIs (this is currently a journal requirement). When p<0.01 please report to 3 decimal places, and when p>/=.01 please report to 2 decimal places.

Thank you for adding a limitation of your study – are there any others? Such as follow-up time post booster, inability to discriminate between omicron lineages etc. Please include all the primary limitations.

Where you report statistical results, please add a space between % symbols and CI i.e. line 55: “(95%CI…”. This is repeated throughout, please check and amend.

**A: We have included the required p-values and fixed the space between CI and % throughout all manuscripts.**

AUTHOR SUMMARY

Thank you for including an author summary. Please ensure it is structured according to PLOS Medicine’s style as described here: <https://journals.plos.org/plosmedicine/s/revising-your-manuscript#loc-author-summary> Specifically, please note the requirement for individually bulleted points and the requirement for the summary to be distinct from the abstract and accessible to the general reader.

**A: We have updated the author summary to fit PLOS Medicine’s style.**

**Author Summary**

**Why Was This Study Done?**

- Brazil and Scotland have been offering boosters for the population that received two doses of vaccines against the coronavirus that causes COVID-19. However, after Omicron (a SARS-CoV-2 variant) emerged, both countries reported a high number of COVID-19 cases despite accelerating their booster programs.
- Knowledge about the duration of the protection offered by the booster doses is essential to guide public health recommendations.

·

**What Did the Researchers Do and Find?**

- We analyzed national databases from Brazil and Scotland between January and April 2022 to estimate the protection offered by mRNA booster doses in individuals who received a primary series of viral vector or mRNA.
- For individuals that received primary series of viral vector vaccine plus mRNA booster, from 14-29 days to ≥ 4 months after the booster, VE against symptomatic infection decreased significantly in Brazil (from 51.6% [95% confidence interval:CI - 51.0 – 52.2] to 4.2% [95% CI 0.7 – 7.6]) and in Scotland (from 67.1% [95% CI 65.5 – 68.5] to 37.4% [95% CI 33.8 – 40.9]). In these periods, a small decrease in VE was observed against severe outcomes in Brazil (from 93.5% [95% CI 93.0 – 94.0] to 82.3% [95% CI 79.7 – 84.7]) and in Scotland (from 98.3% [95% CI 87.3 – 99.8] to 77.8% [95% CI 51.4 – 89.9]). Similar results were obtained with a homologous booster after a primary series of mRNA vaccines.

**What Do These Findings Mean?**

- Modest, short-lived protection was observed against symptomatic infection caused by Omicron variant after two doses of either vector viral or mRNA vaccine plus a booster dose with mRNA vaccine. However, protection against hospitalization or death was substantial for at least three months.
- Similar findings in two very distinct countries allow us to draw reliable results because each country has different key sources of potential bias unrelated to each other.

METHODS and RESULTS

Please quantify main results with p-values as well as 95% CIs (this is currently a journal requirement)

Where p values are reported please also specify the statistical test used to determine it as well as the significance level used (eg p <0.05 two sided)

**A: We have included the required p-values through the text, and included in the methods section that all p-values in the manuscript are two-sided and derived from Wald’s test.**

Line 140: “…for Brazil and Scotland” suggest “in” instead of “for” please revise

**A: We thank the editor for the comment. We have fixed the sentence.**

Line 177: “QCovid risk groups are…” I think it would be helpful to define the QCovid risk before you mention it. As written, it appears as though it is an after-thought and actually, it is vital for the reader to understand how the impact of co-morbidity in this group was accounted for.

It might also be helpful to include the QCovid risk algorithm that you utilized in your analyses in the supporting files – I understand that you have made reference to it but, if possible, it would be helpful to the reader to include it.

**A: We included a brief description of QCOVID the first time we cited it. Also, we included a table with the characteristics evaluated in the algorithm.**

Line 206: “This study is reported following Strengthening….” Please place this statement at the beginning of the methods section

**A: We have moved the sentence as requested.**

Line 209: “The original statistical plan is provided as supplementary appendix.” Suggest “prospective” instead of “original” – please revise throughout because as written, this statement implies that there is a revised analysis plan. Please move this sentence to the beginning of the section entitled “Statistical analysis” Please also indicate where in the appendix this information can be found i.e. S3 as per your manuscript.

**A: We edited the sentence as suggested and included the reference to the supplementary appendix**

In response to point 28 from the previous revision you state the following: “A: All data used in our analysis came from structured administrative databases. No detailed information about the definition of the characteristics was provided.” Please ensure that a relevant sentence is included in your methods section and indicate where you have placed it

**A: We included this information in the methods section.**

“The data in Brazil came from three deterministically linked national structured administrative databases provided by the Ministry of Health: COVID-19 Vaccination Campaign (SI-PNI); Acute Respiratory Infection Suspected Cases (e-SUS-Notifica); and Severe Acute Respiratory Infection/Illness (SIVEP-Gripe). All COVID-19 vaccine doses in Brazil are provided free of charge by the Ministry of Health. All suspected and confirmed cases of COVID-19 must be notified in the e-SUS-Notifica. Regardless of aetiology, all severe acute respiratory illness cases must be notified in the SIVEP surveillance system. Therefore, these three databases should provide 100% country-wide coverage of all notified cases [15]. No detailed information about the definition of comorbidities and race in these databases was provided.”

FIGURES & TABLES

Thank you for including appropriate captions for Figure 1 and Figure 2. In both, instead of “line” suggest “error bars represent 95% Wald confidence interval.”

**A: We have edited the figure caption as suggested.**

Please also include table captions, including for those in the supporting information, which clearly describe the content and include any abbreviations

Please define the numerical values, i.e. n (%), in table column headings (as you have [partially] done for table 1) as opposed to as a footnote – perhaps in the control column?

Table 1 – please remove “N(%)” from the end of the table and include the (%) in the column headings

**A: We moved the “N (%)” from the footnote to the headings: “Characteristics - n(%)”. And we edited all table titles to clearly describe their content.**

In all tables where you report 95% CIs please also report p-values – to conserve space and/or the need for splitting your tables, which are already very full with data, I would consider detailing the p-value significance level in the relevant caption and placing asterisks adjacent to the CIs to reflect the result.

In the table captions please also report the statistical tests used to determine the p-value

**A: We have included symbols to indicate p values (<0.001, </= 0.05, >0.05) for all estimates. And the test used**

Table S2: foot note reads “n (row percent%)” which is confusing to me, please revise for clarity and place this information in column headings (see above)

**A: We have edited the footnote to clarify: “The percentages were calculated using the sum of individuals in each row as the denominator.”**

Table S4 – S8: all titles state the following “Vaccine Effectiveness % (95% CI) against…” suggest for clarity: “ Vaccine Effectiveness reported as percentages (95% CI) against…” or something similar

**A: We have edited the titles of the tables as suggested.**

Figure 1, figure 2, figure S3B and figure S4: Please confirm that this shade of green is selected from a colour palate that this is accessible to those with color blindness – it appears to be a different shade from that used in figure S3A, at least on my monitor!

**A: We have edited the figures to ensure they are colour-blind safe. Now, we are using the Okabe&Ito palette throughout the manuscript.**

Figure S2B: the test is very small and rather inaccessible to the reader, please revise

**A: We have edited the figure to make it readable**

Please remove the data availability statement from the end of the manuscript and please remove the conflict-of-interest statement from the end of the manuscript. This information should be included in the manuscript submission form only and will be complied as metadata

**A: We have removed those items from the manuscript and included them only in the submission form.**

REFERENCES

For in-text reference callouts please add a space between the text and the first of the parentheses. For example line 99: “…wane over time[4,5], prompting…” should read: “…wane over time [4,5], prompting…”. This error is repeated throughout, please check thoroughly and amend

**A: We have fixed the references throughout the manuscript.**
