# Supplementary material for: Effectiveness of mRNA boosters after homologous primary series with BNT162b2 or ChAdOx1 against symptomatic infection and severe COVID-19 in Brazil and Scotland: A test-negative design case–control study
Source: PLoS Med. 2023 Jan 11;20(1):e1004156. doi: 10.1371/journal.pmed.1004156 (PMC9879484; doi:10.1371/journal.pmed.1004156)
Supplement: Attachment — Submitted filename: Requests from Editors_plos.docx [file pmed.1004156.s008.docx]

Requests from Editors:

GENERAL
Thank you for your considerate and detailed responses to previous editor and reviewer requests which we believe have substantially improved your manuscript.
In parts the manuscript reads somewhat as though it has been written by two individuals. This is probably, and understandably, the case given the nature of the study design but it would be beneficial to improve the uniformity of the writing style.

**A**: We have revised the text throughout to make the writing style more consistent.

Please respond to all comments detailed below, in full.
We note the reviewer comments regarding the use of two data sets. The editorial team has discussed this point and have split opinions. The academic editor notes a precedent for the use of two data sets in similar studies and the other reviewers think that the inclusion of the two data sets offer an advance, therefore we conclude that it perhaps amounts to a difference of opinion.

**A**: NA

COMMENTS FROM THE ACADEMIC EDITOR
There is precedent for including multiple datasets in the same manuscript. I've seen it done to compare heterogeneous settings for HIV treatment outcomes. Here the vaccines administered, and SARS-CoV-2 transmission levels, are different across Scotland and Brazil so the comparisons can be useful.

**A**: NA

CONFLICT OF INTEREST STATEMENT
Author Aziz Sheikh sits on the PLOS Medicine Editorial Board. Please add this statement to the manuscript's Competing Interests: "AS is an Academic Editor on PLOS Medicine's editorial board." I could not see that it had been listed.

**A:** We changed the following sentence in the conflict of interest statement “AS is a member of editorial board of PLOS Medicine” to “AS is an Academic Editor on PLOS Medicine’s editorial board”

ABSTRACT
We asked the following: “Thank you for adding a limitation of your study – are there any others? Such as follow up time post booster, inability to discriminate between omicron lineages etc. Please include all the primary limitations” but could not see any amendments, please revise accordingly.

**A:** We have edited the sentence to include the limitation of follow-up time: “Potential limitations of this study were that we assumed that all cases included in the analysis were due to the Omicron variant based on the period of dominance and the limited time of follow-up since the booster dose.”

Line 57 statistical reporting: “37.4% (95% CI 33.8 – 40.9; p<0.001)” thank you for including p-values and CIs suggest reporting as follows “37.4% (95% CI [33.8, 40.9], p<0.001). Please check and amend throughout the abstract, author summary and main manuscript to ensure consistency of reporting

**A:** We have edited the statistical reporting to match the format suggested.

AUTHOR SUMMARY
Line 82: as above, “(from 51.6% [95% confidence interval:CI - 51.0 – 52.2] to 4.2% [95% CI 0.7 – 7.6])” your statistical reporting is rather difficult to interpret, and the use of multiple hyphens can lead to confusion between negative values and intervals.
Suggest the following: “from 51.6%, 95% confidence interval (CI): [51.0, 52.2], to 4.2% [95% CI: 0.7, 7.6])” . Note the absence of the first set of parentheses. Please check and revise throughout
Please include p-values where you report 95% Cis

**A**: We have edited the sentence as suggested

Line 84: sentence beginning “In these periods…” suggest make a separate bulleted for this statement

**A:** We have included an additional bullet point for this sentence.

Line 93: “Similar findings in two very distinct countries allow us to draw reliable results because each country has different key sources of potential bias unrelated to each other”
It would be helpful to clarify “potential bias unrelated to each other”, this statement is vague and unclear to the general reader. In addition, formal comparisons between the two data sets were not made so this statement can’t be fully substantiated by the dataset presented.
I would perhaps reword this statement and consider removing it from the conclusions to the previous section “what did the researchers do and find” as it seems partly aimed at justifying the use of the two data sets.

**A:** We have moved the sentence to “what did the researchers do and find” and edited to “Similar findings in two very different countries allow us to draw reliable results because potential sources of bias in effectiveness studies, such as differences in testing behaviour and unmeasured characteristics between vaccinated and unvaccinated individuals, are unrelated in the two countries.”

METHODS and RESULTS
Please amend statistical reporting as described above

**A:** We have edited the statistical reporting to match the format suggested.

TABLES
Table S2: please define numerical values within and outside of parentheses – suggest “n (%)” as in other tables

**A:** We have included the “n (%)” in the table

FIGURES
Figure S2 B) – please define TND and RT PCR in an appropriate caption

**A:** We have defined TND (Test Negative Design) and RT PCR (Real time polymerase chain reaction) in the figure caption

SOCIAL MEDIA
To help us extend the reach of your research, please provide any Twitter handle(s) that would be appropriate to tag, including your own, your coauthors’, your institution, funder, or lab. Please respond to this email with any handles you wish to be included when we tweet this paper.


Comments from Reviewers:

Reviewer #1: The authors have addressed my points. I still do not see what the added value of having two cohorts presented together is but I seem to be alone in my confusion.

**A**: NA
